# Effectiveness of upgraded maternity waiting homes and local leader training on improving institutional births: a cluster-randomized controlled trial in Jimma, Ethiopia

**DOI:** 10.1186/s12889-020-09692-4

**Published:** 2020-10-22

**Authors:** Jaameeta Kurji, Lakew Abebe Gebretsadik, Muluemebet Abera Wordofa, Sudhakar Morankar, Kunuz Haji Bedru, Gebeyehu Bulcha, Nicole Bergen, Getachew Kiros, Yisalemush Asefa, Shifera Asfaw, Abebe Mamo, Erko Endale, Kednapa Thavorn, Ronald Labonte, Monica Taljaard, Manisha A. Kulkarni

**Affiliations:** 1 School of Epidemiology and Public Health, University of Ottawa, 600 Peter Morand Crescent, Ottawa, Ontario K1G 5Z3 Canada; 2 Department of Health, Behaviour & Society, Jimma University, Jimma Town, Jimma Zone Ethiopia; 3 Department of Population & Family Health, Jimma University, Jimma Town, Jimma Zone Ethiopia; 4 Jimma Zone Health Office, Jimma Town, Jimma Zone Ethiopia; 5 Faculty of Health Sciences, University of Ottawa, 600 Peter Morand Crescent, Ottawa, Ontario K1G 5Z3 Canada; 6 Department of Health Economics, Management & Policy, Jimma University, Jimma Town, Jimma Zone Ethiopia; 7 Ottawa Hospital Research Institute General Campus, University of Ottawa, Ottawa, Canada; 8 Ottawa Hospital Research Institute Civic Campus, University of Ottawa, Ottawa, Canada

**Keywords:** Cluster-randomized controlled trial, Complex interventions, Maternity waiting home, Institutional birth, Ethiopia, Maternal healthcare, Community engagement, Three-delays model, RE-AIM framework

## Abstract

**Background:**

Maternity waiting homes (MWHs), residential spaces for pregnant women close to obstetric care facilities, are being used to tackle physical barriers to access. However, their effectiveness has not been rigorously assessed. The objective of this cluster randomized trial was to evaluate the effectiveness of functional MWHs combined with community mobilization by trained local leaders in improving institutional births in Jimma Zone, Ethiopia.

**Methods:**

A pragmatic, parallel arm cluster-randomized trial was conducted in three districts. Twenty-four primary health care units (PHCUs) were randomly assigned to either (i) upgraded MWHs combined with local leader training on safe motherhood strategies, (ii) local leader training only, or (iii) usual care. Data were collected using repeat cross-sectional surveys at baseline and 21 months after intervention to assess the effect of intervention on the primary outcome, defined as institutional births, at the individual level. Women who had a pregnancy outcome (livebirth, stillbirth or abortion) 12 months prior to being surveyed were eligible for interview. Random effects logistic regression was used to evaluate the effect of the interventions.

**Results:**

Data from 24 PHCUs and 7593 women were analysed using intention-to-treat. The proportion of institutional births was comparable at baseline between the three arms. At endline, institutional births were slightly higher in the MWH + training (54% [*n* = 671/1239]) and training only arms (65% [*n* = 821/1263]) compared to usual care (51% [*n* = 646/1271]). MWH use at baseline was 6.7% (*n* = 256/3784) and 5.8% at endline (*n* = 219/3809). Both intervention groups exhibited a non-statistically significant higher odds of institutional births compared to usual care (MWH^+^ & leader training odds ratio [OR] = 1.09, 97.5% confidence interval [CI] 0.67 to 1.75; leader training OR = 1.37, 97.5% CI 0.85 to 2.22).

**Conclusions:**

Both the combined MWH^+^ & leader training and the leader training alone intervention led to a small but non-significant increase in institutional births when compared to usual care. Implementation challenges and short intervention duration may have hindered intervention effectiveness. Nevertheless, the observed increases suggest the interventions have potential to improve women’s use of maternal healthcare services. Optimal distances at which MWHs are most beneficial to women need to be investigated.

**Trial registration:**

The trial was retrospectively registered on the Clinical Trials website (https://clinicaltrials.gov) on 3rd October 2017. The trial identifier is NCT03299491.

## Background

Maternity waiting homes, which are temporary residential facilities within or close to health facilities, have been used to improve pregnant women’s access to skilled obstetric care for almost seven decades [1] in an effort to stem maternal mortality rates. MWHs may be of particular interest in sub-Saharan Africa where the level of maternal mortality was still highest in the world in 2017 (542 maternal deaths per 100,000 livebirths) [2].

In 2016, institutional births in Ethiopia stood at just 26% with substantial variation occurring between regions [3]. Several barriers to accessing maternal healthcare services are experienced by women [4, 5]; chief among them are geographical [6–10] and social factors [5, 11–13]. MWHs typically target women experiencing geographical barriers to accessing obstetric care and those with a high risk of delivery complications.

In 2011, there were nine MWHs in Ethiopia located in faith-based or non-governmental organization health facilities [14]; by 2016, over half of the national facilities surveyed were providing waiting services [15]. Despite the integration of MWHs as part of national efforts to improve maternal and child health [16], their effectiveness has not been evaluated in a trial setting [17]. Observational studies from Zimbabwe comparing MWH users to non-users that have reported favourable pregnancy [18] and neonatal [19, 20] outcomes among MWH users. In Ethiopia specifically, a retrospective, hospital-based cohort study using 2011–2014 data reported lower odds of stillbirths (OR = 0.18, 95% CI: 0.13 to 0.25) and a lower number of maternal deaths (0% vs. 0.3%) among MWH users compared to non-users [21].

Levels of MWH utilization globally have been reported to be sub-optimal, partly due to the poor quality of services available at MWHs [17]. A recent Zambian study using upgraded MWHs reported increased utilization levels at one of the two improved sites [22]. Social support for pregnant women has also been found to impact MWH use [23]. Women often require family and neighbours to assist with childcare [24, 25, 27] and household chores while they are away; in instances where food is not provided, MWH users require family assistance in supplying meals. Women’s absence may also result in loss of family income requiring support from their husbands [14, 28]. Community support is particularly important in the Ethiopian context where MWHs rely on community contributions for their construction and operation [16] and could influence use [28].

In light of the evidence gap concerning the effectiveness of MWHs to improve institutional birth levels, two intervention components were developed: upgraded MWHs to provide quality services, and training for local religious and community leaders to create an enabling environment for women and their families to access MWHs and obstetric care. The objective of the trial was to evaluate the effectiveness of upgraded MWHs and local leader training in improving institutional births in Jimma Zone, Ethiopia.

## Methods

The trial protocol has been published previously [29] and the trial was retrospectively registered on 3rd October 2017 with Clinical Trials (trial identifier: NCT0329949).

### Setting

**Fig. 1:**
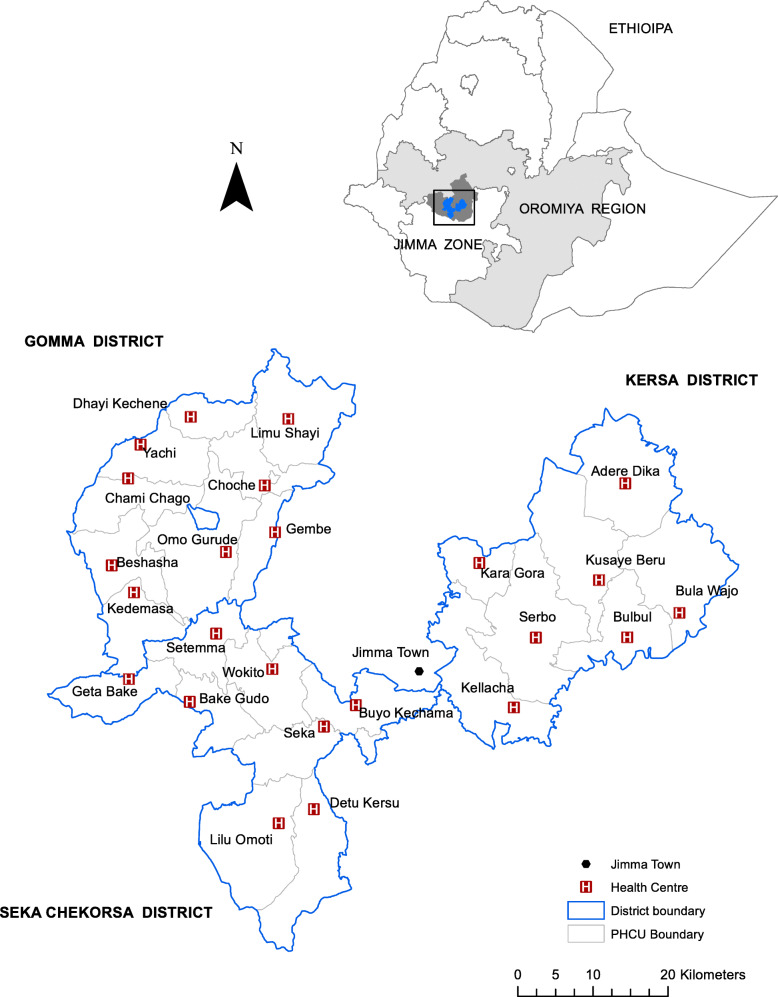
Map of study districts depicting locations of health centres and Jimma Town (created using ArcMap version 10.6.1 Redlands, CA: Environmental Systems Research Institute, Inc.)

The trial was conducted within Gomma, Seka Chekorsa and Kersa districts in Jimma Zone, Oromiya region (Fig. 1). Together, the districts had about 153,000 households in 2015/2016 [30]. Jimma town situated roughly in the centre of the three districts is about 350 km from the capital, Addis Ababa.

Women typically receive maternal healthcare services at primary health care unit (PHCU) level; PHCUs comprise a health centre and satellite health posts that are each operated by community-based health extension workers (HEWs). Health posts serve populations of up to 5000 by providing preventive and basic curative services. Jimma Zone has eight hospitals, 122 health centres and 566 health posts [31]. HEWs function as important links between the community and the health system by referring women to health centres for antenatal and obstetric care and providing follow up postnatal care (PNC). They are often supported by the Women’s Development Army (WDA) whose members are women regarded as leaders in their communities for successfully adopting the health and sanitation guidelines outlined in the Health Extension Program packages [32]. Health centres are usually staffed with clinical officers and midwives; in 2016, 21 of the 24 study health centres had one or two midwives trained in basic emergency obstetric care (BEmOC) [30].

### Design

A pragmatic, three-arm, stratified, cluster-randomized trial design was used to evaluate the effect of upgraded, functional MWHs (MWH^+^) and leader training on the primary outcome of institutional births. The trial arms consisted of: (i) upgraded MWH^+^ combined with religious and community leader training (“local leader training”) around safe motherhood strategies to mobilize communities; (ii) local leader training alone; and, (iii) usual care. PHCU catchment areas served as clusters, were randomized to trial arms, and were the level at which the interventions were delivered.

PHCUs were eligible to participate in the trial if maternity waiting services were available at the health centre. Women of reproductive age who reported a pregnancy outcome (livebirth, stillbirth, induced or spontaneous abortion) 12 months prior to each round of survey were eligible for inclusion in cross-sectional surveys at baseline and 21 months post intervention roll-out [29]. Women who experienced induced/spontaneous abortions were not excluded as they could benefit from the leader training intervention activities and seek maternal healthcare services. In order to detect an absolute difference in the proportion of institutional births of 0.17 with 80% power, 24 clusters with 160 women each (assuming equal cluster sizes) were required for each round of surveys. This assumed a control arm proportion of 0.4 and used a two-sided alpha of 0.025 to account for two pairwise comparisons [29]. Using the method described by Hooper and Bourke, the product of two design effects were used to inflate the sample size required under a similarly powered individually randomized design. The first design effect, due to cluster randomization, was calculated using a within-period intracluster correlation coefficient (ICC) of 0.1 [33]; the second design effect, due to repeated assessments, was calculated using the within-period ICC and a cluster autocorrelation coefficient of 0.8 which allowed for a 20% decay in strength of the ICC among women surveyed in different time periods [34].

To ensure a balanced distribution of poorly functioning MWHs and health centres with low capacity to provide BEmOC, stratified randomization was used as described previously [29]. Briefly, using 2016 Jimma Zone Health Office (JZHO) data on MWH functionality, MWHs were classified as high functioning (≥ 5 service indicators present) or low functioning (< 5 service indicators present). BEmOC capacity was classed as high (≥5 of the 7 signal functions present) or low (< 5 signal functions present). Clusters were grouped into the four strata that resulted and a random number generator in STATA was used to create the allocation sequence [26]. The allocation sequence was made known to the study coordinator in May 2017 when distribution of MWH supplies to intervention sites began; this was also when health centre staff at MWH + leader training sites were made aware of their allocation status.

Data collectors identified randomly pre-selected households, screened women for eligibility, provided information (survey objectives, institutions involved, expectations from participants, participant rights, and risks and benefits of participating), answered questions and took verbal consent from women wishing to be interviewed. About 4% of women interviewed at endline were also interviewed during baseline as no exclusions were made based on prior participation.

### Usual care

The level of existing services is described in the trial protocol [29]. Briefly, MWHs are modelled as government-community partnerships and rely on cash or in-kind contributions from the community for construction and operation. There was considerable variation in quality across the MWHs in the study area. In 2016, 16 of the 24 waiting facilities were poorly functioning lacking basic items such as bedding, cooking utensils, a reliable water supply or electricity [30]. Women generally depend on HEW or midwives referrals to access MWHs and referral practices differed between sites.

HEWs are mostly responsible for conducting health promotion activities within the community and are aided by the WDA. Religious leaders are acknowledged to be influential members of the community and formative work revealed that they consider promoting access to maternal healthcare services and providing support to pregnant women part of their role [35]. However, there is little evidence of how widespread or consistent efforts by religious leaders are to promote institutional births and/or use of MWHs in the study districts.

### Interventions

The MWH^+^ intervention component entailed upgrading and standardizing existing waiting facilities based on minimum needs identified through formative evaluation [29] and guided by the national policy [16] to create a home-like environment for pregnant users. MWHs situated at health centres in intervention arms were equipped with bedding, utensils and personal hygiene items, solar lamps, water tanks, drinking water purifiers, cooking stoves and cleaning items. Supplies were transported to intervention sites under the auspices of the district health offices. Remuneration for an MWH attendant to cook and clean was also provided. A register was also introduced to better track users [29]. In order to avoid disrupting the community contribution systems used to support MWHs particularly with food provision, no meals were supplied through the study. During the first month after supply distribution, the study coordinator visited intervention sites to ensure appropriate setup of materials at the MWHs and to brief midwives on correct completion of the MWH register placed at interventions MWHs. However, after this time supportive supervision visits were part of the agreed-upon role of the Jimma Zone Health Office and the District Health Offices. This strategy was employed to test out and facilitate sustainable mechanisms of MWH operation.

In recognition of the fact that women’s social environments are as important in influencing use of maternal healthcare services [36, 37] as individual-level factors such as education [38, 39] and service quality, the local leader training intervention component was created. HEWs, religious leaders and community leaders (members of the WDA) attended workshops that facilitated identification of access barriers to maternal healthcare services. HEWs were all women with at least a secondary school education and were between 20 and 30 years of age. WDA members generally reflected the demographic profile of women in the community. Religious leaders from the two major religious groups in the area (Christian and Muslim) were mostly male, had completed some level of primary school and were between 30 and 50 years of age. Building on their experiences, participants were encouraged to identify strategies to support their communities in overcoming these barriers to make motherhood safer and to promote use of antenatal care, MWHs, delivery care at facilities and postnatal care. Due to the pragmatic nature of the trial, the commitment to community empowerment and establishment of sustainable practices, leaders were encouraged to create activities they felt were optimal for their communities. Positive strategies used to improve access to services identified through formative research (such as urging women’s social networks to assist with childcare and domestic chores, encouraging family and friends to accompany pregnant women to health facilities or working together to organize transport for women) [35] were discussed during training as part of brainstorming locally suited strategies for leaders to promote. HEWs committed to co-facilitating the WDA workshops, revamping pregnant women conferences to discuss safe motherhood and use of MWHs with women and collaborating with religious leaders to improve community contributions to MWHs. Religious leaders opted to address safe motherhood strategies during their religious gatherings and attend any community events organized by HEWs and WDAs to promote use of maternal healthcare services or tackle access challenges faced by the community.

### Blinding

**Fig. 2:**
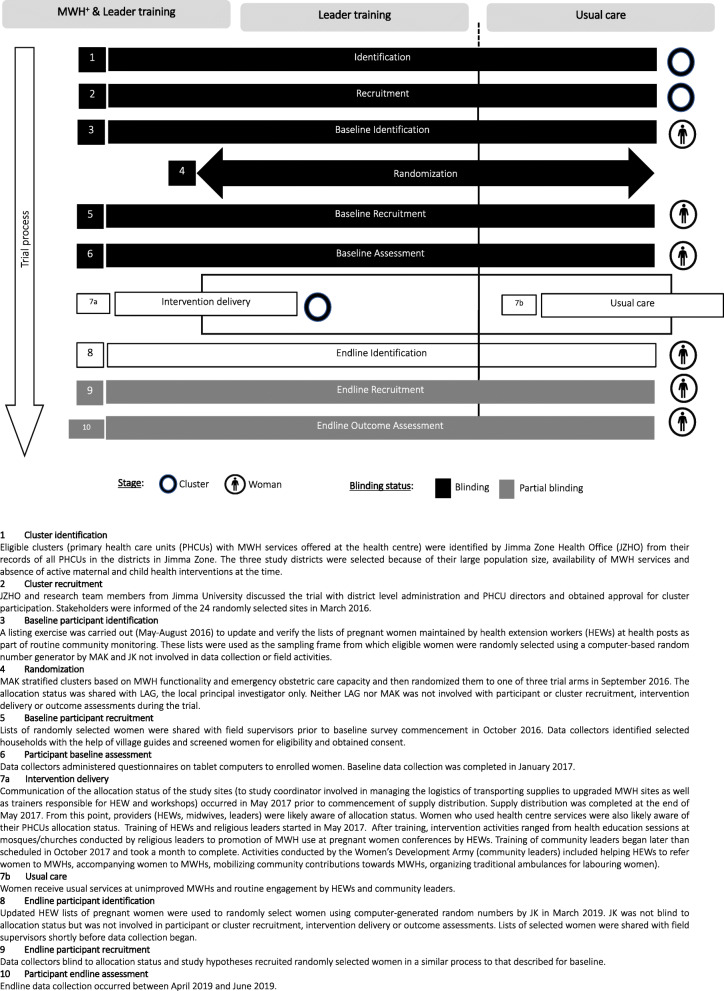
Timeline cluster diagram illustrating participant recruitment, randomization, outcome assessments and blinding status of the trial

Data collectors were blind to women’s allocation status during both baseline and endline assessments. It was not possible to blind women or healthcare providers to intervention status but both groups were unaware of the study hypotheses. Figure 2 outlines the trial processes depicting order of participant recruitment, randomization, intervention delivery and outcome assessments; blinding status is indicated using black for complete blinding and grey for partial blinding. This timeline cluster tool is recommended for assessing risk of bias in cluster randomised trials [40].

Identification and recruitment of clusters as well as identification of women for the baseline survey occurred prior to randomization, making identification bias unlikely. Once MWHs upgrading was completed and leader activities commenced, providers (health centre staff, HEWs) and participants (women) across the study area may have been aware of their cluster’s allocation status depending on the extent of their interaction with health centres and each other. The risk of contamination through leaders in the control arm encouraging their congregation to deliver at health facilities, as well as performance bias from being aware of allocation to the intervention arms cannot be precluded.

### Data collection and outcome measures

The primary outcome was institutional birth defined as delivery of the last child at a health facility where obstetric care is provided (i.e health centre or hospital) as reported by an enrolled woman. Secondary outcomes included antenatal care (self-reported antenatal care received for last child delivered) and postnatal care (self-reported postnatal care received for last child delivered within 48 h and 6 weeks). Outcomes were measured at baseline and 21 months after the introduction of interventions (“endline”) through household surveys. Data were collected using interviewer-administered questionnaires in Afaan Oromo or Amharic which contained sections on socio-demographics, reproductive history, attitudes towards and use of maternal health care service use including MWHs, danger sign knowledge and social support. The questionnaire was adapted based on the Ethiopia Demographic Health Survey [41] and the JHPIEGO birth preparedness and complication readiness monitoring tool kit [42]; the MWH module was developed by the research team. (Supplement 1).

### Protocol amendments

The protocol specified that endline data collection would take place 24 months after the introduction of the interventions. However, due to delays in intervention rollout experienced due to political instability in the country, endline outcomes were assessed after a shorter duration of intervention exposure. Similarly, resource and time constraints necessitated the cancellation of an additional round of data collection (midline survey). This resulted in the need to increase the minimum absolute detectable difference in institutional births from 15% as planned, to 17% to maintain prespecified sample size and power.

### Analysis

An intention to treat approach was used for primary analysis where original cluster assignments to trial arms were maintained regardless of whether interventions were delivered or not. Institutional births were compared at endline between intervention and control groups using a generalized linear mixed model. The model included a random intercept for PHCU to account for within-period ICC as well as a random cluster-period effect to account for the between-period ICC [34]. Differences at baseline were constrained by including fixed effects for time and intervention by time. The Kenward-Roger degrees of freedom approximation was used to account for bias due to the relatively small number of clusters included in the trial [43]. Secondary outcomes, namely postintervention antenatal care and postnatal care use, were analysed as described for the primary outcome. Odds ratios with 97.5% confidence intervals (two-sided alpha of 0.025 used) were used to report comparisons between intervention groups and control. The ICCs for outcomes were calculated on the proportions scale. Data analysis was conducted in STATA version 15 and SAS version 9.4.

Although not specified in the trial protocol, frequency tables, descriptive statistics and graphs were generated to contextualize the findings on the impact of the interventions on institutional births. The intervention components were expected to increase the levels of institutional births by improving awareness and use of functional MWHs and enhancing women’s access to facility obstetric care by mobilizing community support in tackling barriers. Thus, four main areas were explored: (i) awareness of MWHs (ii) appropriate linkage of women to MWHs (iii) use of MWHs and obstetric services and (iv) quality of MWH services as part of ancillary analyses.

## Results

### Baseline characteristics of participants

**Fig. 3:**
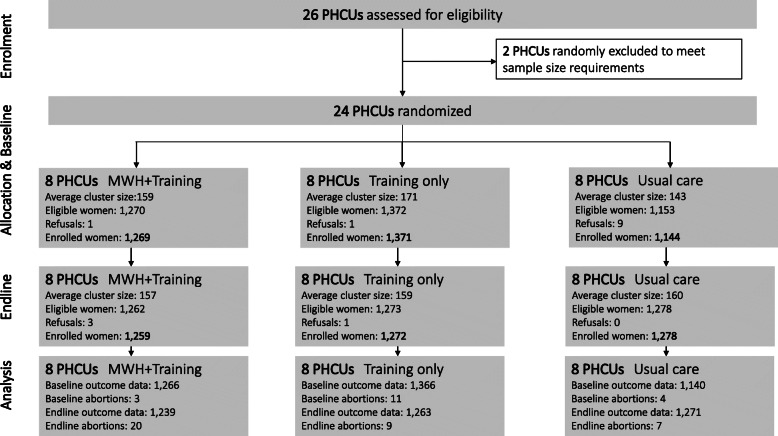
CONSORT participant flow diagram

All 24 randomly selected PHCUs received their respective treatment allocations and were included in the analysis (Fig. 3). The average observed cluster sample size at baseline ranged from 143 in control PHCUs to 171 in training only PHCUs. This difference was partially due to the replacement of ineligible women from one PHCU with pre-selected replacements from other PHCUs when replacements ran out. Data on institutional births were available for all enrolled women at baseline and endline apart from those who had abortions as pregnancy outcomes.

Slightly less than 50% of women in the study area PHCUs had some level of education; most women were housewives and had more than one child (Table 1). Their husbands generally engaged in farming and about half had a primary school education. The majority of households reported being within an hour of a health facility. Clusters were comparable across most characteristics. Clusters in the leader training arm had a slightly higher proportion of educated women when contrasted with the MWH^+^& training or usual care arms. ANC and PNC use as well as institutional birth levels in leader training PHCUs was also slightly higher than the other two groups but this difference was not statistically significant (data not shown). Poorly functional MWHs were also mainly found in the MWH^+^& training and usual care arms.

**Table 1:** Baseline characteristics of clusters and individuals by trial arm

Participant characteristics	MWH + training (*n* = 1269)		Training only (*n* = 1371)		Usual care (*n* = 1144)		Overall (*n* = 3784)	
**Cluster level**								
Mean PHCU sample size (Standard deviation)	159 (15)		171 (41)		143 (20)		158 (29)	
PHCU sample size range	136–189		116–242		111–166		111–242	
	n	(%)	n	(%)	n	(%)	n	(%)
Educated women	550	(44)	677	(50)	456	(40)	1683	(45)
Least poor households	593	(47)	569	(43)	351	(31)	1513	(40)
Poor functioning MWH	6	(75)	3	(38)	7	(88)	16	(67)
≥1 BEmOC trained midwife	6	(75)	8	(100)	7	(88)	21	(88)
HEW home visit	418	(32.9)	477	(34.8)	375	(32.8)	1270	(33.6)
Antenatal care use	1052	(82.9)	1215	(88.6)	911	(79.6)	3178	(84.0)
Maternity waiting home use	98	(7.7)	88	(6.4)	70	(6.1)	256	(6.8)
Institutional births	608	(48.0)	726	(53.2)	519	(45.5)	1853	(49.1)
Postnatal care use	491	(38.8)	576	(42.2)	421	(36.9)	1488	(39.4)
**Individual level**								
Women’s age	n	(%)	n	(%)	n	(%)	n	(%)
< 20 years	84	(6.9)	102	(7.6)	62	(5.6)	248	(6.8)
20–30 years	790	(65.0)	862	(64.1)	705	(63.5)	2357	(64.2)
> 30 years	342	(28.1)	381	(28.3)	344	(31.0)	1067	(29.0)
Women’s occupation								
Housewives	980	(77.2)	1064	(77.6)	890	(77.8)	2934	(77.5)
Other	289	(22.8)	307	(22.4)	254	(22.2)	850	(22.5)
Parity								
1 child	274	(21.6)	329	(24.0)	224	(19.6)	827	(21.9)
> 1 child	995	(78.4)	1042	(76.0)	920	(80.4)	2957	(78.1)
Husband’s education level								
None	562	(47.0)	508	(38.9)	506	(46.5)	1576	(43.9)
Primary	530	(44.3)	659	(50.5)	493	(45.3)	1682	(46.9)
Secondary/higher	104	(8.7)	138	(10.6)	89	(8.2)	331	(9.2)
Husband’s occupation								
Farmer	982	(82.2)	1085	(83.4)	950	(87.6)	3017	(84.3)
Other	212	(17.8)	216	(16.6)	135	(12.4)	563	(15.7)
Travel time to health centre^a^								
< 1 h	914	(75.7)	1059	(79.3)	854	(78.4)	2827	(77.9)
≥ 1 h	293	(24.3)	276	(20.7)	235	(21.6)	804	(22.1)

^a^The majority of women reported walking to health centres (approx. 88%). About 10% used motorized transport while the rest relied on bicycles or animals

### Post-intervention institutional births

The proportion of institutional births in the study area increased across all groups between baseline and endline (Table 2). While both the combined intervention (OR = 1.09, CI: 0.67 to 1.75) and training alone (OR = 1.37, CI: 0.85 to 2.22) slightly improved institutional births compared to usual care, the increases were not statistically significant.

**Table 2:** Effectiveness of interventions on improving institutional births and secondary outcomes (ANC, PNC)

	MWH^**+**^ &Training n (%)		Training only n (%)		Usual care n (%)		Overall n (%)	
**Institutional births**	(B) *n* = 1266		(B) *n* = 1366		(B) *n* = 1140		(B) *n* = 3772	
	(E) *n* = 1239		(E) *n* = 1263		(E) *n* = 1271		(E) *n* = 3773	
Baseline births	608 (48.0)		726 (53.2)		519 (45.5)		1853 (49.1)	
Endline births	671 (54.2)		821 (65.0)		646 (50.8)		2138 (56.7)	
Odds ratio^a^ (97.5% CI)	1.09 (0.67 to 1.75)		1.37 (0.85 to 2.22)		Reference			
ICCs	Within-period ICC = 0.1098							
	Between period ICC = 0.0912							
	Cluster autocorrelation coefficient = 0.831							
**Antenatal care**	(B) n = 1269		(B) *n* = 1371		(B) *n* = 1144		(B) *n* = 3784	
	(E) *n* = 1259		(E) *n* = 1272		(E) *n* = 1272		(E) *n* = 3809	
Baseline use	1052	(82.9)	1215	(88.6)	911	(79.6)	3178	(84.0)
Endline use	1081	(85.9)	1176	(92.5)	1056	(82.6)	3313	(87.0)
Odds ratio (97.5% CI)	0.99 (0.59 to 1.66)		1.38 (0.80 to 2.38)		Reference			
ICCs	Within-period ICC = 0.0764							
	Between period ICC = 0.0624							
	Cluster autocorrelation coefficient = 0.816							
**Postnatal care**	(B) *n* = 1266		(B) *n* = 1366		(B) *n* = 1141		(B) *n* = 3773	
	(E) *n* = 1239		(E) *n* = 1263		(E) *n* = 1271		(E) *n* = 3773	
Baseline use	491	(38.8)	576	(42.2)	421	(36.9)	1488	(39.4)
Endline use	526	(42.5)	649	(51.4)	564	(44.4)	1739	(46.1)
Odds ratio (97.5% CI)	0.88 (0.55, 1.39)		1.05 (0.66, 1.67)					
ICCs	Within-period ICC =0.0828							
	Between period ICC = 0.0678							
	Cluster autocorrelation coefficient = 0.819							

*B* Baseline, *E* Endline

^a^Odds ratio refers to between-arm difference controlling for baseline

Increases between baseline and endline were also noted for both ANC and PNC use. However, there was no difference in ANC use between PHCUs in the combined intervention group compared to usual care (OR = 0.99, CI: 0.59 to 1.66). The increased ANC use in the training only group compared to usual care was not statistically significant (OR = 1.38, CI: 0.80 to 2.38). Neither interventions had a significant effect on PNC use.

### Ancillary analyses

#### Awareness about MWH services and benefits

**Fig. 4:**
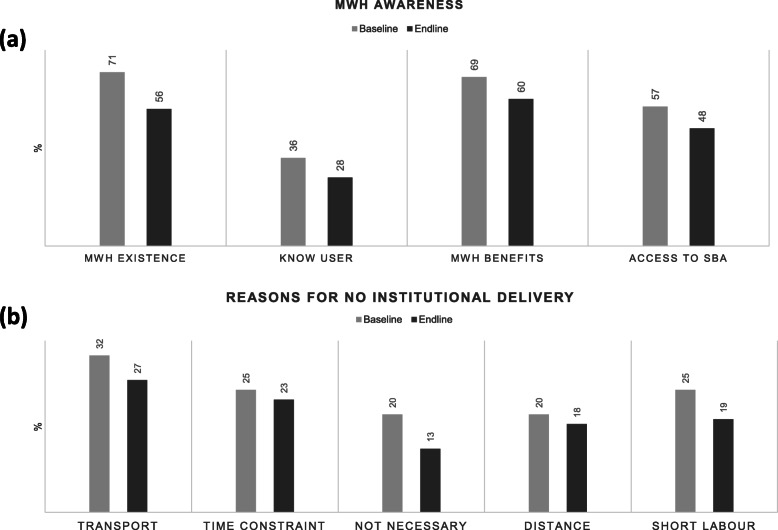
Bar charts of (**a**) dimensions of MWH awareness among women (**b**) reasons for no institutional delivery

As shown in Fig. 4a, awareness about the existence of MWHs, knowing someone who had used the services, awareness of benefits associated with MWH stay and women’s ability to link MWH stay with easier access to skilled obstetric care was lower in endline compared to baseline; however, there were no significant differences between trial arms during either survey round. HEWs and nurses were reported to be sources of information for over 50% of women surveyed during endline. Less than 1% of women relied on WDA for health information (such as danger signs during pregnancy) and none of the women surveyed cited religious leaders as a source. HEW contact with families through home visits was similar between baseline and endline (34% vs. 32%) and was not significantly different between trials arm at endline.

#### Linking women to MWHs

Hardly any women interviewed during the surveys reported getting an MWH referral as part of their birth preparedness planning. During endline, over 75% of MWHs users were referred by HEWs or midwives in intervention arms compared to about half in the control arm but the difference was not statistically significant (*p*-value 0.097). Obtaining a referral represented about 32% of responses to questions about reasons for MWH stay and was significantly higher in the MWH^+^& training arm (47%) compared to usual care (12%, *p*-value 0.0292). Expecting complications (34%), large distances between home and health facility (33%) and needing rest (23%) formed a large part of other reasons for MWH stay.

#### Use of MWH and obstetric services

Overall, MWH use was higher at baseline (6.7%, *n* = 256/3784) than endline (5.8%, *n* = 219/3809). MWH utilization in the MWH^+^& training arm was low with less than five women surveyed reporting MWH use in five of the eight PHCUs. Moreover, there was no significant difference in MWH use (p-value 0.6343) at endline between the intervention (MWH^+^+training 6.4% *n* = 80/1259; training 4.6% *n* = 58/1272) and control arms (6.3%, *n* = 81/1278). About 40% of non-users cited a lack of awareness as their reason for not using an MWH while living too close to need one was described by 27% of non-users during endline; neither reasons were significantly different between trial arms. Nevertheless, four intervention sites had over 55% of non-users reporting a lack of awareness about MWHs. Reasons related to concerns around quality of services, prior bad experiences or hearing bad reviews from other women were uncommon (1–4%). Similarly, access-related issues such as high transport costs (< 1%) and social factors like no childcare (4%) or getting resistance from family members (2%) were described by only a small proportion of non-users.

Overall, 51% (*n* = 1919/3772) women at baseline gave birth at a health facility; this increased to 57% (*n* = 1635/3773) at endline. The most commonly cited barrier among women during both survey rounds (Fig. 4b) was lack of transport, followed by time constraints and large distances between facilities and homes. Almost half the women who mentioned distance as a barrier were unaware of MWH services. The proportion of women who felt giving birth at facilities was unnecessary because they were healthy or had previous successful home births dropped from 20% in baseline to 13% in endline. However, only the transport barrier was significantly different between trial arms (34% MWH^+^&training, 21% training arm, 27% usual care) at endline (*p*-value 0.0384). About 50% of respondents during both baseline and endline reported having a health centre within or close to their area of residence, but there was no significant difference between trial arms. However, there were significant differences between clusters during both periods. During baseline 40% of women reported walking to the health facility for their last delivery, 30% relied on ambulance services and 22% used some form of motorized transport; during endline the use of motorized transport increased to 31%, but there was no significant difference between trial arms in mode of transport at endline (*p*-value 0.6845). The fraction of women living with 30 min of a health facility was similar between baseline and endline (71% vs 74%); and although in endline a slightly higher proportion lived more than 30 min away in the MWH^+^& training (31%) and control arms (28%) compared to the leader training only arm (20%), the difference was not significant (p-value 0.0689).

**Fig. 5:**
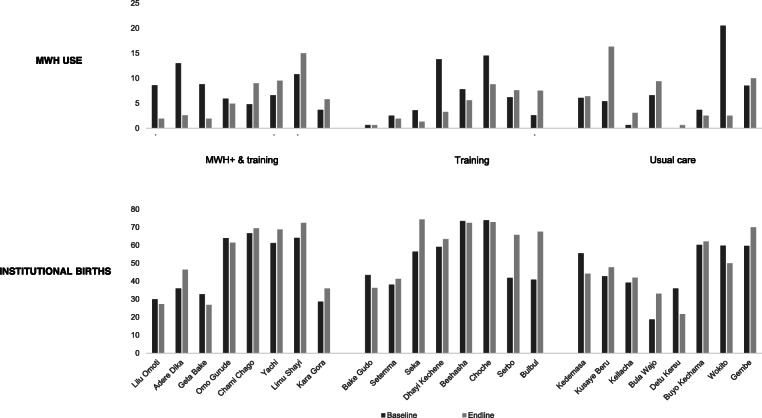
Bar chart of MWH use and institutional births across PHCUs and over survey periods

While increases in MWH use generally coincided with increases in institutional births across all three arms (Fig. 5), a few PHCUs experienced increases in institutional births while MWH use declined between baseline and endline. In Adere Dika (MWH^+^&training arm) for instance, MWH use dropped by 10% but institutional births increased by 10%; over 40% of non-users reported short distances to health facilities as their reason for not choosing to use MWHs. A similar response was also observed in two PHCUs in the training-only arm (Setemma and Seka).

#### Quality of services

Satisfaction with health facilities in general appeared to be slightly higher during baseline than endline (positive ratings: 82% vs. 73%); the differences in quality ratings between trial arms during endline (74% MWH^+^& training, 78% training and 68% control) were statistically significant (*p*-value 0.0434). However, while the majority of MWH^+^& training MWHs were reported to have provided beds/bedding and food or cooking facilities, very few users were checked on by midwives (21%), had access to toilets and clean water (16%) or bathrooms (14%). Despite this, all MWH^+^& training users said they would recommend MWH stay to other women. Notably, Kusaye Beru in the control arm registered high performance in terms of number of services received by users.

## Discussion

The combination of upgraded MWHs and leader training lead to small but non-significant improvements in institutional birth levels in Jimma Zone. One reason for this lack of effect may have been low exposure to the interventions due to security concerns in the country during the trial period. A state of emergency was declared in Ethiopia in 2016 and in 2018 [44, 45]. Political unrest, particularly in Oromiya region, may have hampered leaders’ abilities to conduct planned activities and women’s safe access to interventions. This may partially explain the lack of difference in awareness of MWH services and benefits across trial arms although intervention arms would have been expected to have higher awareness level. Higher community acceptance and use of MWHs in Ethiopia has been credited to better awareness about MWH services and associated benefits [14]. WDA community leaders may also have experienced personal challenges in conducting community activities as research from Amhara region found that WDAs had poor living conditions, suffered from food insecurity and debt and were not able to access the government resources they hoped for when joining the WDA [46]. The voluntary nature of the WDA has also been reported to hamper efforts by HEWs who often rely on WDAs to link women to services [47]. The consideration of MWHs as part of birth preparedness planning was very low, suggesting a need to better promote MWHs as a means to overcome distance and transport barriers. Family Conversations, for instance, conducted by HEWs to engage families to better prepare for delivery [48] would be a useful platform to broaden conventional delivery plans which traditionally focus on saving money for transport and calling ambulances.

In this context women relied heavily on HEW referrals to gain entry to MWHs. Referral activities appeared to be significantly higher in the intervention arms compared to control suggesting HEWs participating in the intervention arms were encouraging women to use MWHs. Still, it was likely that referral activities had not reached optimal levels with only one-third of women reporting home visits by HEWs across all three arms at endline. This could mean that women who were not able to come to a health post for services may not have been referred to MWHs by HEWs; a study on how HEWs use their time at work reported they spend 25% of their day waiting for clients at the health post and about 35% on administrative tasks and travel [49]. Despite only 12% of non-users in this trial citing no referrals as the reason for not using an MWH, more investigation is needed on HEW referral practices; in particular, to whom HEWs promote MWHs to since they are the most common source of health information for women and an important link to health services in this setting and elsewhere in Ethiopia [50]. Data on compliance to MWH referrals by women is also needed to distinguish between low use due to deficient referral levels versus minimal compliance. A study in Kenya reporting low MWH use recommended follow up of women referred to MWHs as high referral rates were not matching utilization levels [51].

Low MWH use has often been linked to the poor quality of services offered. Only 15% of women in endline from the MWH + training arm who did not use MWHs said it was because they were dissatisfied with the quality of services. The majority of MWH users in this arm reported receiving bedding and meals. However, access to toilets, water and bathrooms was low. Monitoring of MWH users by midwives was also reported by just one-third of users. While midwives were briefed on use of MWH registers and their continued role in referring women to MWHs, they were not provided additional training as part of the intervention. While it is unlikely that upgraded MWHs increased delivery workloads substantially as reported in other settings [52], more engagement with midwives may have improved levels of MWH user monitoring.

Another important reason for low MWH use among some women may have been a relative short distance between homes and health facilities making direct access to the facility possible. Travel time and distance have been reported to be inversely correlated with MWH use [23, 53]. Almost half of the women in endline who had not used an MWH said they lived close to the facility. Almost three-quarters of women reported living within 30 min of a health facility which could possibly make MWHs as a solution to physical inaccessibility unnecessary for them. Despite this, distance as a barrier to delivering at a health facility persists as a reason for delivering at home. There is, therefore, a need to establish how far from health facilities women need to reside for MWHs to be most beneficial to them to better gauge unmet need.

### Strengths and limitations

One of the main strengths of the trial and intervention design were that they adopted a pragmatic approach to reflect conditions in which interventions were intended for use outside of a research setting [54]. Using an “integrative ecological paradigm” recognizes that community interventions are a part of “larger complex systems” and can “disrupt or enhance existing community resources”; they generally aim to expand local capacity [55]. Intervention design in this trial focused on improving existing MWHs without diminishing established community contributions. Engaging HEWs as co-facilitators for leader training built on roles as community leaders, critical links between the community and the health system and trusted sources of information [35, 56]. Indeed, empowering religious leaders and WDA members to design engagement activities leveraged their influence within the communities while reinforcing their leadership roles. Partnering with Jimma Zone administration led to interventions designed not only to incorporate end-user preferences but also included improvements that were aligned with what policymakers also expected could be to be feasibly scaled.

The improvement in outcomes across all arms including usual care made it difficult to detect an improvement due to the interventions, compounded by the fact that the difference was smaller than anticipated. Without examination of process data generated through qualitative and project monitoring, it is difficult to assess the extent of intervention delivery. A limitation in this paper, therefore, is the inability to distinguish between small effect due to implementation issues resulting in lower intervention exposure and an ineffective intervention. While the security situation reduced the team’s ability to conduct all planned monitoring visits, the available data will be analysed separately to assess the range of activities conducted by trained local leaders to understand why the training-only arm had an unexpectedly higher effect that the combined intervention. Both monitoring and qualitative data sources will also be useful in uncovering any co-interventions that may have occurred and diminished the intervention’s effects as well as shed light on contextual factors that could explain the patterns in MWH use and institutional births observed between PHCUs. It will be interesting to see if increases in institutional births in PHCUs with declines in MWH use were as a result of locally created solutions that improved access to delivery care but did not require women to be absent from their homes a few weeks before delivery.

A second important limitation was the relatively short duration of intervention exposure that may have been insufficient enough to observe any significant changes in the communities and in women’s behaviours. Complex interventions, that do not necessarily exhibit linear causality from input to outcome and engage “active agents” with adaptable behaviours, often require several years of implementation time [57]. In fact, a review on evaluating the effectiveness of behaviour change techniques described trial timescales as typically being at least 3 years long [58].

Finally, if a specific distance cut-off had been included in the participant eligibility criteria, it may have focused the evaluation on women who may be experiencing geographical barriers. However, the distance relevant to this setting has yet to be established given the large proportion of households located within an hour of a health facility.

## Conclusions

While only a small effect on institutional births was found from introducing upgraded MWHs and training local leaders, the trial findings point to the importance of integrating engagement of communities and health workers along with quality improvements to MWHs. Without improved community awareness and support for MWHs and the presence of effective referral mechanisms to link women to these services, quality improvements may be insufficient to improve utilization rates and ultimately increase institutional births. Moreover, though flexibility in intervention activities is important for tailoring solutions to local circumstances, consistent supportive supervision may be needed to encourage successful delivery of interventions.

## Supplementary information

**Additional file 1.** Copy of the questionnaire used in the trial.

## Data Availability

Data used for this analysis will be provided by the authors upon reasonable request to the principal investigator, Dr. Manisha Kulkarni (manisha.kulkarni@uottawa.ca).

## Abbreviations

ANC: Antenatal care
BEmOC: Basic emergency obstetric care
CI: Confidence interval
HEW: Health extension worker
ICC: Intracluster correlation coefficient
JZHO: Jimma Zone Health Office
MWH: Maternity Waiting Homes
OR: Odds ratio
PHCU: Primary health care unit
PNC: Postnatal care
WDA: Women’s Development Army
