# Supplementary material for: Effectiveness of upgraded maternity waiting homes and local leader training on improving institutional births: a cluster-randomized controlled trial in Jimma, Ethiopia
Source: BMC Public Health. 2020 Oct 22;20:1593. doi: 10.1186/s12889-020-09692-4 (PMC7583173; doi:10.1186/s12889-020-09692-4)
Supplement: Supplementary file 1 — Additional file 1. Copy of the questionnaire used in the trial. [file 12889_2020_9692_MOESM1_ESM.pdf]

Evaluation of Interventions to Promote Safe Motherhood in Jimma Zone, Ethiopia
QUESTIONNAIRE FOR INDEX WOMAN

**INTERVIEWER: Complete this section before approaching household**					
**CP1**	Interview date	USE ETHIOPIAN CALENDAR<br>☐☐/☐☐/☐☐☐☐<br>D D M M Y Y Y Y		**CP16** Survey phase	
**CP2**	Interviewer ID	☐☐		**CP3** Screening ID	7-DIGITS<br>☐☐☐☐☐☐☐
**CP4**	Woman's first name			**CP5** Woman's last name	
**CP17**	District	¹○ Gomma	²○ Seka Chekorsa	³○ Kersa	
**CP20**	Survey site (PHCU)	ENTER PHCU WHERE TEAM IS<br>**GOMMA**<br>¹○ Beshasha<br>²○ Chami Chago<br>³○ Choche<br>⁴○ Dhayi Kechene<br>⁵○ Gembe<br>⁶○ Kedemasa<br>⁷○ Limu Shayi<br>⁸○ Omo Gurude<br>⁹○ Yachi	**SEKA CHEKORSA**<br>¹○ Bake Gudo<br>²○ Buyo Kechema<br>³○ Detu Kersu<br>⁴○ Geta Bake<br>⁵○ Lilu Omoti<br>⁶○ Seka<br>⁷○ Setemma<br>⁸○ Wokito	**KERSA**<br>¹○ Adere Dika<br>²○ Bala Wajo<br>³○ Bulbul<br>⁴○ Kusaye Beru<br>⁵○ Kara Gora<br>⁶○ Kellacha<br>⁷○ Serbo	
**CP6**	Kebele of residence	**SEKA CHEKORSA**<br>☐ Kusaro<br>☐ Buyo Qacamaa<br>☐ Andode Allaggee<br>☐ Gibe Bosoo<br>☐ Meexii<br>☐ Ushane Koche<br>☐ Gura Ula'ukke<br>☐ Shashamanne<br>☐ Magala Saqqaa<br>☐ Dabo Yaya<br>☐ D/Gibee<br>☐ B/Rogee	☐ Ilke Tunjo<br>☐ Wokito Medaaluu<br>☐ Xeebo Waacho<br>☐ Deto Qarssu<br>☐ BidaruTuulii<br>☐ Ushane Buyo<br>☐ Qoraa Waacoo<br>☐ Meti Ushaane<br>☐ Gudoo Daakaa<br>☐ Komo Haarri<br>☐ Lili Ca'aa<br>☐ Nasee	☐ Siba Qaqee<br>☐ Budo Keraa<br>☐ Ilke Togobee<br>☐ Bake Gudo<br>☐ Atro Sufaa<br>☐ Dimtu Shekota<br>☐ Gaxa Bakke<br>☐ Geppa Sadan<br>☐ Sakala Genefoo<br>☐ Sogido<br>☐ Sentema Goroo<br>☐ Geshe Lucine	
		**GOMMA**<br>☐ Beshasha<br>☐ Bore dinsira<br>☐ Keda maye<br>☐ Omo Funtule<br>☐ Kotta<br>☐ Kedemasa<br>☐ Getabore	☐ Bulado Choche<br>☐ Koye Sejja<br>☐ Limu Shaye<br>☐ Limu Sapha<br>☐ Keta Bero<br>☐ Acha Afeta<br>☐ Meti Koticha	☐ Gomma 2<br>☐ Gembe<br>☐ Omo Gobu<br>☐ Belfo Konche<br>☐ Keso Hiti<br>☐ Yachi<br>☐ Kilole	

		**GOMMA continued**<br>☐ Barsoma<br>☐ Elbu<br>☐ Dalecho<br>☐ Chedero Suse<br>☐ Bulbulo<br>☐ Omo Gurude<br>☐ Omo Beko	☐ Tesso Sadecha<br>☐ Dedessa<br>☐ Gomma 1<br>☐ Choche<br>☐ Choche Lemmi<br>☐ Dinu<br>☐ Gabene Abo	☐ Dedo Ureche<br>☐ Gogga Kamise<br>☐ Dhayi kechene<br>☐ Chami Chago<br>☐ Jimate Deru<br>☐ Odo Adami
**CP6 cont.**	Kebele of residence (continued)	**KERSA**<br>☐ Awaye Sebu<br>☐ Babo<br>☐ Tikur Balto<br>☐ Tikur Abulo<br>☐ Girma<br>☐ Serbo<br>☐ Toli Kersu<br>☐ Adere Dika<br>☐ Folla Gubeta<br>☐ Mara Kebericho	☐ Ankeso<br>☐ Gunju<br>☐ Kombolcha<br>☐ Merewa<br>☐ Kojja Mujja<br>☐ Bulbul<br>☐ Gello<br>☐ Kitimbile<br>☐ Wadiko<br>☐ Kusaye Beru<br>☐ Kellacha	☐ Busa Bechane<br>☐ Kersa sume<br>☐ Bala wajo<br>☐ Dogoso<br>☐ Osso<br>☐ Siba<br>☐ Sinkulle<br>☐ Kara Gora<br>☐ Shewa Totoba<br>☐ Gora Seriti
**INTERVIEWER: Introduce yourself and follow consent procedures before enrolling woman**				
**CNST1**	Woman eligible?	*INTERVIEWER: ask woman if she gave birth to a child, had a stillbirth or miscarriage/abortion during the past 12 months.*<br><br>1○ Yes<br>0○ No SKIP TO END (Report to supervisor)		
**CP8**	Consent obtained?	1○ Yes	0○ No SKIP TO END. Fill REFUSAL form	
**CP10**	Household ID	5-DIGITS<br>☐☐☐☐☐	**CP9**<br>Index woman ID	6-DIGITS<br>☐☐☐☐☐ 1
**CP11**	Is woman's name correct?	1○ Yes SKIP TO CP12	0○ No	
**CP11a**	Woman's correct first name		**CP11b**<br>Woman's correct last name	
**CP12**	Husband's first name		**CP13**<br>Husband's last name	
**CP7**	Household GPS	Latitude<br>☐☐☐°☐☐'☐☐"	Longitude<br>☐☐☐°☐☐'☐☐"	

**CP18**	Name of health centre woman attends	**GOMMA**<br>1○ Beshasha<br>2○ Chami Chago<br>3○ Choche<br>4○ Dhayi Kechene<br>5○ Gembe<br>6○ Kedemasa<br>7○ Limu Shayi<br>8○ Meti Koticha<br>9○ Omo Gurude<br>10○ Yachi	**SEKA CHEKORSA**<br>1○ Bake Gudo<br>2○ Buyo Kechema<br>3○ Dabo Yaya<br>4○ Detu Kersu<br>5○ Geta Bake<br>6○ Lilu Omoti<br>7○ Seka<br>8○ Setemma<br>9○ Wokito	**KERSA**<br>1○ Adere Dika<br>2○ Bala Wajo<br>3○ Bulbul<br>4○ Kusaye Beru<br>5○ Kara Gora<br>6○ Kellacha<br>7○ Serbo
**CP19**	Husband present for interview	1○ Yes	0○ No	

Adapted from: Demographic and Health Surveys, JHPIEGO Birth preparedness/complication readiness tools for MNH & EQ-5D-3L Health Questionnaire Euro Qol Group 2009 (UK)

SECTION 1: [DEM] SOCIODEMOGRAPHIC INFORMATION

Good morning/afternoon. My name is ______________________. Thank you for taking the time to speak with me. I would like to start by asking you some general questions about yourself and your family.

**DEM1a.**	Do you know the year and month you were born in?	1○ Yes 0○ No SKIP TO DEM3
**DEM1.**	What year were you born in?<br><br>*INTERVIEWER: Use the Calendar of events if needed. Record answers in Ethiopian calendar.*	☐☐☐☐<br>Y Y Y Y
**DEM2.**	What month were you born in?<br><br>*INTERVIEWER: Record answers in Ethiopian calendar.*	☐☐ SKIP TO DEM5 if complete<br>M M<br>99○ Do not know
**DEM3.**	***If year of birth not known***<br>What is your age?	☐☐ years
**DEM5.**	Have you ever attended school?	1○ Yes 0○ No SKIP TO DEM7
**DEM6.**	What is the highest level of education you have completed?	0○ None<br>1○ Grade 1<br>2○ Grade 2<br>3○ Grade 3<br>4○ Grade 4<br>5○ Grade 5<br>6○ Grade 6<br>7○ Grade 7<br>8○ Grade 8<br>9○ Grade 9<br>10○ Grade 10<br>11○ Grade 11<br>12○ Grade 12<br>13○ Higher<br>88○ Other (*specify*)

**DEM7.**	Now I'd like you to read these four sentences to me.<br><br>***INTERVIEWER Show literacy card in preferred language to respondent.***<br><br>*Probe: Can you read any part of these sentences to me?*	0○ Cannot read at all<br>1○ Able to read some parts of the sentences<br>2○ Able to read all four sentences in full<br>3○ Visually impaired literate<br>4○ Visually impaired non-literate
**DEM8.**	What work do you mainly do?	1☐ Housewife<br>2☐ Student<br>3☐ Farmer<br>4☐ Trader<br>5☐ Government employee<br>6☐ Private organization employee<br>7☐ Domestic worker<br>8☐ Daily labourer<br>88☐ Other (*specify*)
**DEM9.**	Have you worked in the last 12 months?	1○ Yes<br>0○ No
**DEM10.**	What is your marital status?	1○ Never married SKIP TO DEM18<br>2○ Married<br>3○ Divorced<br>4○ Separated<br>5○ Widowed SKIP TO DEM18<br>88○ Other (*specify*)
**INTERVIEWER: Ask questions about husband if NOT PRESENT to be interviewed** (see response in CP19)		
**DEM11a**	Do you know the year and month your husband was born in?	1○ Yes<br>0○ No SKIP TO DEM13
**DEM11.**	What year was your husband born in?<br><br>*INTERVIEWER: Record answers in Ethiopian calendar.*	☐☐☐☐<br>Y Y Y Y
**DEM12.**	What month was your husband born in?<br><br>*INTERVIEWER: Record answers in Ethiopian calendar.*	☐☐<br>M M<br>99○ Do not know
**DEM13.**	***If year of birth not known***<br><br>How old is your husband?	☐☐ years<br>99○ Do not know
**DEM14.**	What **kind of work** does your husband mainly do?	1☐ Student<br>2☐ Farmer<br>3☐ Trader<br>4☐ Government employee<br>5☐ Private organization employee<br>6☐ Domestic worker<br>7☐ Daily labourer<br>88☐ Other (*specify*)

**DEM15.**	Has your husband **worked** in the last 12 months?	1○ Yes 0○ No 99○ Do not know
**DEM16.**	Has your husband **ever attended school**?	1○ Yes 0○ No SKIP TO DEM18 99○ Do not know SKIP TO DEM18
**DEM17.**	What is the **highest level of education** your husband has completed?	0○ None<br>1○ Grade 1<br>2○ Grade 2<br>3○ Grade 3<br>4○ Grade 4<br>5○ Grade 5<br>6○ Grade 6<br>7○ Grade 7<br>8○ Grade 8<br>9○ Grade 9<br>10○ Grade 10<br>11○ Grade 11<br>12○ Grade 12<br>13○ Higher<br>99○ Do not know<br>88○ Other (*specify*)
**DEM18.**	How many individuals are part of your household?<br><br>(*i.e usually stay together and have shared arrangements for eating, cooking and pool their money together)*	**INTERVIEWER:** DO NOT FORGET TO INCLUDE ALL CHILDREN<br><br>☐☐
**DEM19.**	Of these, how many household members are **adults** (18 years or above)?	☐☐
**DEM20.**	How many of the adults in the household have **jobs or are earning money** through work or trade?	☐☐

SECTION 2: [IDM] INFORMATION SOURCES & HEALTH-RELATED DECISION MAKING

I would now like to ask you some questions about your sources of information and how decisions are made in your household.

**IDM1.**	How often do you read a **newspaper or magazine -** not at all, at least once a week or more than once a week?	0○ Not at all	1○ At least once a week	2○ More than once a week
**IDM2.**	How often do you listen to the **radio -** not at all, at least once a week or more than once a week?	0○ Not at all	1○ At least once a week	2○ More than once a week
**IDM3.**	How often do you watch **television -** not at all, at least once a week or more than once a week?	0○ Not at all	1○ At least once a week	2○ More than once a week

**IDM4.**	Do you own a mobile phone?	1○ Yes 0○ No
**IDM5.**	Where do you usually get health information from?<br><br>*Probe: Any other place?*	1☐ Newspaper/Magazine<br>2☐ Radio<br>3☐ Television<br>4☐ Nurse/doctor<br>5☐ Health extension worker<br>6☐ Husband<br>7☐ Relative<br>8☐ Friends/neighbours<br>9☐ Traditional birth attendant<br>10☐ Health Development Army member<br>88☐ Other (*Specify*)
**IDM6.**	What were your main sources of advice/information about **where to deliver** your last child?<br><br>*Probe: Any other place?*	1☐ Newspaper/Magazine<br>2☐ Radio<br>3☐ Television<br>4☐ Nurse/doctor<br>5☐ Health extension worker<br>6☐ Husband<br>7☐ Relative<br>8☐ Friends/neighbours<br>9☐ Traditional birth attendant<br>10☐ Health Development Army member<br>88☐ Other (*Specify*)
**IDM7.**	Has a health extension worker (HEW) ever visited your home?	1○ Yes 0○ No SKIP TO IDM11
**IDM8.**	How often does the HEW visit your home?	0○ Less than once a month<br>1○ 1-2 times a month<br>2○ 3-4 times a month (or every week)<br>3○ 5 or more times a month
**IDM9.**	Does the HEW provide health information during her visits?	1○ Yes 0○ No SKIP TO IDM11
**IDM10.**	What information does the HEW share with you during her visits to your home?<br><br>*INTERVIEWER: Do not read out. Select all that apply.*	1☐ Antenatal care at health facilities<br>2☐ Care during pregnancy (diet, hygiene, rest, vaccinations, etc)<br>3☐ Danger signs during pregnancy<br>4☐ Birth/safe delivery planning<br>5☐ Danger signs during labour

	Probe: Anything else?	6☐ Postpartum danger signs in mother<br>7☐ Danger signs in newborn<br>8☐ Newborn care – feeding, immunizations, cord care,etc<br>88☐ Other (*Specify*)	
**IDM11.**	Did the HEW visit you after you delivered your last child?	1○ Yes	0○ No SKIP TO IDM13
**IDM12.**	How many times did the HEW visit you in the 6 weeks (42 days) after you had given birth to your last child?<br><br>*INTERVIEWER: Double check your numbers to ensure that they make sense.*	☐☐	99○ Do not recall
**IDM13.**	In the **last 12 months**, have you or anyone in your family participated in or joined a programme that promotes antenatal care, delivery at health facilities or after birth care for mother and baby at health facilities?	1○ Yes	0○ No SKIP TO IDM25
**IDM24**	What programs have you or your family participated in, in the last 12 months?		
**IDM25**	Have you been interviewed before for the IMCHA study like we are doing today?<br><br>*INTERVIEWER: You can remind the participant what this study is about and that researchers from Jimma University and Ottawa University in Canada are*	1○ Yes	0○ No

	conducting the study and visited the districts between October 2016 and January 2017.	
**IDM14.**	Who usually decides how the money **you earn** will be used?	1○ Self<br>2○ Husband<br>3○ Jointly with husband<br>4○ Mother<br>5○ Father<br>6○ Mother-in-law<br>7○ Father-in-law<br>88○ Other (*Specify*)
**IDM15.**	*INTERVIEWER: Skip if woman not married.*<br><br>Who usually decides how the money **your husband** earns will be used?	1○ Self<br>2○ Husband<br>3○ Jointly with husband<br>4○ Mother<br>5○ Father<br>6○ Mother-in-law<br>7○ Father-in-law<br>88○ Other (*Specify*)
**IDM16.**	Who usually makes decisions about **health care** for **yourself**?	1○ Self<br>2○ Husband<br>3○ Jointly with husband<br>4○ Mother<br>5○ Father<br>6○ Mother-in-law<br>7○ Father-in-law<br>88○ Other (*Specify*)
**IDM17.**	Who usually makes decisions about **health** care for your **children**?	1○ Self<br>2○ Husband<br>3○ Jointly with husband<br>4○ Mother<br>5○ Father<br>6○ Mother-in-law<br>7○ Father-in-law<br>88○ Other (*Specify*)
**IDM18.**	Who usually decides whether or not you will use **family planning** or birth spacing methods?	1○ Self<br>2○ Husband<br>3○ Jointly with husband<br>4○ Mother<br>5○ Father<br>6○ Mother-in-law<br>7○ Father-in-law<br>88○ Other (*Specify*)
**IDM19.**	Who made the **final** decision about where you would give birth to your **last** child?	1○ Self<br>2○ Husband<br>3○ Jointly with husband<br>4○ Mother<br>88○ Other (*Specify*)

		5○ Father<br>6○ Mother-in-law<br>7○ Father-in-law	
**IDM20.**	Who usually decides whether you can **leave the house** to visit family/friends, go out shopping, etc?	1○ Self<br>2○ Husband<br>3○ Jointly with husband<br>4○ Mother<br>5○ Father<br>6○ Mother-in-law<br>7○ Father-in-law	88○ Other (*Specify*)
**IDM21.**	Are you a member of any social group, organization or association?<br><br>For example, Women's Development Army, or iddir, etc?	1○ Yes	0○ No SKIP TO REP1
**IDM22.**	What group are you a member of?<br><br>***INTERVIEWER: Select all that apply***	1☐ Farmers' group<br>2☐ Women's group<br>3☐ Youth group<br>4☐ Kebele committee<br>5☐ Credit/savings group like *iddir, ekub*	6☐ Community Conversation<br>7☐ NGO-led group<br>8☐ Health Development Army<br>88☐ Other (*Specify*)
**IDM23.**	Are you a leader of the women's health development army?	1○ Yes	0○ No

SECTION 3: [REP] REPRODUCTIVE HISTORY OF INDEX WOMAN

I'd now like to ask you some questions about any pregnancies and births you have had during your life.

**REP1.**	How old were you when you first got married?<br><br>INTERVIEWER: *If woman cannot remember, ask how long she has been married for and help her work out her age when she first got married.*	☐☐ years
**REP2.**	How old were you when you first got pregnant?	☐☐ years
**REP3.**	How many times have you been pregnant during your life?	☐☐

**REP6**	How many **livebirths** have you **ever** had?	☐☐	*INTERVIEWER*: *Check against total pregnancies and other pregnancy outcomes*
**REP4**	How many times in your life have you had an **induced abortion**?	☐☐	*INTERVIEWER*: *Check against total pregnancies and other pregnancy outcomes*
**REP5**	How many times in your life have you had a **miscarriage**?	☐☐	*INTERVIEWER*: *Check against total pregnancies and other pregnancy outcomes*
**REP7**	How many times in your life have you had a **stillborn child**?	☐☐	*INTERVIEWER*: *Check against total pregnancies and other pregnancy outcomes*

**INTERVIEWER RESPONSE CHECK #1**	**Total pregnancies (REP3)**	☐☐ COPY FROM REP3
**Do total pregnancies match total pregnancy outcomes?**<br><br>Enter totals in columns to double check responses in REP3-REP7	Total livebirths (REP6)	☐☐ (1) COPY FROM REP6
	Total abortions (REP4)	☐☐ (2) COPY FROM REP4
	Total miscarriages (REP5)	☐☐ (3) COPY FROM REP5
	Total stillbirths (REP7)	☐☐ (4) COPY FROM REP7
	**Total pregnancy outcomes**	☐☐ (1+2+3+4)
	REP6check. Did the woman ever have twins?	1○ Yes 0○ No

**REP8**	How many children have you delivered **at home**?	☐☐
**REP9**	How many children have you delivered at a **health centre or hospital**?	☐☐
**REP10**	How many children have you delivered **en route** (ex: on the road, in the ambulance)?<br><br>*INTERVIEWER: This is the number of deliveries that did not occur at the respondent's home or at the health facility but elsewhere. For example: on the way to the health centre, at a relative's home, TBA's home, etc.*	☐☐

**INTERVIEWER RESPONSE CHECK #2**	Total livebirths (REP6)	☐☐ (1) COPY FROM REP6
**Do total births match total delivery sites reported?**<br><br>*INTERVIEWER: Enter totals in columns to double check responses in REP8-REP10 against REP6-REP7*	Total stillbirths (REP7)	☐☐ (2) COPY FROM REP7
	**Total births**	☐☐ (1+2)
	Total home births (REP8)	☐☐ (3) COPY FROM REP8
	Total facility births (REP9)	☐☐ (4) COPY FROM REP9

		Total births en route (REP10)	☐☐ (5) COPY FROM REP10
		**Total delivery sites**	☐☐ (3+4+5)
**REP14.**	Did your **last pregnancy** result in a livebirth, stillbirth, miscarriage or abortion?	1○ Live birth – full term<br>2○ Live birth – preterm<br>3○ Still birth	4○ Miscarriage<br>5○ Abortion
**REP15.**	Have you ever given birth to a **premature baby** (i.e gave birth to a live baby before completing 37 weeks of pregnancy)?	1○ Yes	0○ No
**REP16.**	Did you plan your last pregnancy?	1○ Yes	0○ No
**REP17.**	During your **last pregnancy**, did you experience any **serious health problems** related to the pregnancy?	1○ Yes	0○ No SKIP TO REP22
**REP18.**	What SERIOUS health problems did you experience during your last pregnancy?<br><br>***Select all that apply***	1☐ Bleeding<br>2☐ Severe headache<br>3☐ Blurred vision<br>4☐ Convulsions/fits<br>5☐ Swollen face/hands<br>6☐ High fever<br>7☐ Loss of consciousness/fainting<br>8☐ Breathing difficulty<br>9☐ Severe weakness<br>10☐ Severe abdominal pain<br>11☐ More/less fetal movement<br>12☐ Water breaks without labour<br>13☐ Persistent vomiting	14☐ Infection<br>15☐ Mental health problems (ex: depression)<br>88☐ Other(*Specify*)
**REP19.**	Did you **get help**/seek assistance for the problem(s)?	1○ Yes	0○ No SKIP TO REP21
**REP20.**	ASK THIS QUESTION IF **REP19=Yes** Then GO TO REP22<br><br>Where did you get help from?	1☐ Husband<br>2☐ Family/relative<br>3☐ Friends/neighbours<br>4☐ Health extension worker<br>5☐ Doctor/nurse	88☐ Other (*Specify*)

	Select all that apply	6☐ Traditional birth attendant (TBA)<br>7☐ Health Development Army
**REP21.**	ASK THIS QUESTION IF **REP19=No** Then GO TO REP22<br><br>What was the **reason for not** seeking assistance for the problem (s)?<br>***Select all that apply***	1☐ Didn't think it was necessary<br>2☐ Husband/family didn't think it was necessary<br>3☐ Facility too far<br>4☐ No transport<br>5☐ No childcare<br>6☐ Too expensive<br>7☐ Poor quality services<br>8☐ Used home remedy<br>9☐ Didn't know where to go<br>10☐ Had no time<br>11☐ Long wait times<br>12☐ Inconvenient hours<br>13☐ Felt better<br>14☐ Health post usually closed<br>88☐ Other (*Specify*)
**REP22.**	Did you suffer from any of these conditions during your last pregnancy?<br><br>***INTERVIEWER: Read out all the health conditions listed.***	0☐ No<br>1☐ High blood pressure<br>2☐ Diabetes<br>3☐ HIV<br>4☐ Malaria<br>5☐ Other infection
**REP23.**	Have you experienced any health problems related to pregnancy during your **other previous pregnancies**?	1○ Yes<br>0○ No SKIP TO HSU1
**REP24.**	What health problems did you experience during your other previous pregnancies?<br><br>***INTERVIEWER: Select all that apply***	1☐ Bleeding<br>2☐ Severe headache<br>3☐ Blurred vision<br>4☐ Convulsions/fits<br>5☐ Swollen face/hands<br>6☐ High fever<br>7☐ Loss of consciousness/fainting<br>8☐ Breathing difficulty<br>9☐ Severe weakness<br>14☐ Infection<br>15☐ Mental health problems (ex: depression)<br>88☐ Other(*Specify*)

		10☐ Severe abdominal pain<br>11☐ More/less fetal movement centre<br>12☐ Water breaks without labour<br>13☐ Persistent vomiting

SECTION 4: [HSU] MATERNAL HEALTH CARE UTILIZATION

I would now like to ask you some questions about health services that you may have used during your last pregnancy, during your delivery of your last child and after delivery.

4.1 Antenatal care

**HSU1.**	Have you ever received antenatal care for any of your **previous pregnancies**?	1○ Yes	0○ No SKIP TO HSU3
**HSU2.**	Where did you usually receive antenatal care during your previous pregnancies?<br><br>***Select all that apply***<br><br>*INTERVIEWER: Select the place woman went most often for ANC care. Enter all sites mentioned in "Other" if it is not clear which site was used most often.*	1☐ Health post<br>2☐ Health centre<br>3☐ Maternity waiting home<br>4☐ Hospital<br>88☐ Other (*Specify*)	
**HSU3.**	Did you see anyone for antenatal care during your **last pregnancy**?	1○ Yes SKIP TO HSU5	0○ No
**HSU4.**	SKIP TO HSU16 WHEN COMPLETE<br><br>What was the **reason for not getting antenatal care** during your last pregnancy?	1☐ Didn't think it was necessary<br>2☐ Husband/family didn't think it was necessary<br>3☐ Facility too far<br>4☐ No transport<br>5☐ No childcare<br>6☐ Too expensive<br>7☐ Poor quality services<br>8☐ Used home remedy	13☐ Felt better<br>14☐ Health post usually closed<br>88☐ Other (*Specify*)

		9☐ Didn't know where to go<br>10☐ Had no time<br>11☐ Long wait times<br>12☐ Inconvenient hours		
**HSU5.**	SKIP IF HSU3=NO<br>How many times did you visit the health facility for antenatal care during your **last pregnancy**?	☐☐	0○ Do not remember	
**HSU6.**	SKIP IF HSU3=NO<br><br>During your **last pregnancy**, how many months pregnant were you when you went for your **FIRST** antenatal care visit at the health facility?	☐☐ months	0○ Do not remember	
**HSU7.**	SKIP IF HSU3=NO<br><br>How many months pregnant were you when you **LAST** received antenatal care for your **last pregnancy**?	☐☐ months	0○ Do not remember	
**HSU8.**	SKIP IF HSU3=NO<br><br>Where did you **mainly** receive antenatal care from during your last pregnancy?<br><br>*<u>INTERVIEWER</u>: Select the place woman went most often for ANC care. Enter all sites mentioned in "Other" if it is not clear which site was used most often.*	1○ Own home<br>2○ Someone's home<br>3○ Government hospital<br>4○ Government health centre<br>5○ Government health post<br>6○ Private hospital<br>7○ Private clinic	88○ Other (*Specify*)	
**HSU12.**	SKIP IF HSU3=NO<br><br>Did you receive **counselling** as part of the antenatal care visit during your last pregnancy?	1○ Yes	0○ No SKIP TO HSU16	99○ Do not remember SKIP TO HSU16
**HSU13.**	SKIP IF HSU3=NO<br><br>Can you tell me **what information** you were provided with during the counselling session?<br><br>***Do not prompt.***	1☐ Antenatal care at health facilities<br>2☐ Care during pregnancy (diet, hygiene, rest, vaccinations, etc)<br>3☐ Danger signs during pregnancy	9☐ Newborn care – feeding, immunizations<br>88☐ Other (*Specify*)	

	Select all that apply	4☐ Birth/safe delivery planning<br>5☐ MWH services<br>6☐ Danger signs during labour<br>7☐ Postpartum danger signs in mother<br>8☐ Danger signs in newborn
**HSU14.**	SKIP IF HSU3=NO<br><br>Did the health worker recommend where you should consider delivering your baby?	1○ Yes<br>0○ No SKIP TO HSU16<br>98○ Do not remember SKIP TO HSU16
**HSU15.**	SKIP IF HSU3=NO<br><br>Where did the health worker recommend you should deliver your baby?	1○ Health post<br>2○ Health centre<br>3○ Hospital<br>4○ Home<br>98○ Do not remember<br>88○ Other (*Specify*)

4.2 Intrapartum care

**HSU16.**	Where have you **usually** given birth to your children in the past?<br><br>*INTERVIEWER: Select the place where most deliveries occurred. Enter all sites mentioned in "Other" if it is not clear which site was used most often.*	1○ Own home<br>2○ Someone else's home (ex: relative, TBA)<br>3○ Government hospital<br>4○ Government health centre<br>5○ Government health post<br>6○ Private hospital<br>7○ Private clinic<br>88○ Other (*Specify*)
**HSU17.**	SKIP to WDK1 IF REP14=miscarriage or abortion<br><br>Where did you give birth to your **last child**?	1○ Own home SKIP TO HSU19<br>2○ Someone else's home (ex: relative, TBA)<br>3○ Government hospital<br>4○ Government health centre<br>5○ Government health post<br>6○ Private hospital<br>7○ Private clinic<br>88○ Other (*Specify*)

**HSU18.**	How did you reach the place where you gave birth to your **last child**?	1○ By foot<br>2○ By taxi<br>3○ Bajaj (motorbike rickshaw)<br>4○ Local stretcher<br>5○ Ambulance<br>6○ By horse/mule<br>7○ By bicycle<br>88○ Other (*Specify*)
**HSU19.**	SKIP TO HSU20 IF HSU17=hospital, health centre or clinic<br><br>***INTERVIEWER: Ask only if respondent did NOT deliver at a health facility***<br><br>What were the reasons why you did not give birth to **your last child** at a health facility?<br><br>***Select all that apply***	1☐ Didn't think it was necessary<br>2☐ Husband/family didn't think it was necessary<br>3☐ Facility too far<br>4☐ No transport<br>5☐ No childcare<br>6☐ Too expensive<br>7☐ Poor quality services<br>8☐ Unexpected/short labour<br>9☐ Didn't know where to go<br>10☐ Had no time<br>11☐ No privacy<br>12☐ Inconvenient hours<br>13☐ Fear of procedures<br>14☐ Wanted family present<br>15☐ Not comfortable receiving services from male health care workers<br>16☐ Preferred birthing position not allowed at health facility<br>88☐ Other (*Specify*)
**HSU20.**	Did you plan to give birth to your **last child** at this place?	1○ Yes<br>0○ No
**HSU21.**	Prior to the delivery of your **last child** did you or your family make ANY **arrangements for the birth** of the child?	1○ Yes<br>0○ No SKIP TO HSU47
**HSU22.**	What did you do? Did you…….<br><br>***<u>INTERVIEWER</u>: Read out options and select all that apply***	1☐ Save money for delivery?<br>2☐ Organize transport to delivery location?<br>3☐ Identify skilled delivery attendant?<br>4☐ Get an MWH referral?<br>5☐ Identify blood donor?<br>9☐ Identify a health facility to go to in case of emergency?<br>88☐ Other (*Specify*)

		6☐ Identify someone to look after your home?<br>7☐ Familiarize yourself with your estimated delivery date?<br>8☐ Identify a birth companion?	
**HSU47**	Prior to the delivery of your last child did you make a **plan for an emergency or complication** during pregnancy?	1○ Yes	0○ No SKIP TO HSU23
**HSU48**	What did you do? Did you…..<br><br>***INTERVIEWER*: Read out options and select all that apply**	1☐ Save money for an emergency?<br>2☐ Identify someone to look after your home during the emergency?<br>3☐ Identify a health facility to go to in case of emergency?<br>4☐ Organize transport to a health facility in case emergency?	88☐ Other (*Specify*)
**HSU23.**	Who assisted with the delivery of your **last child**?<br><br>***INTERVIEWER*: Do NOT prompt. Select all that apply**	1☐ Husband<br>2☐ Family/relatives<br>3☐ Friends/neighbours<br>4☐ Health extension worker<br>5☐ Doctor/nurse<br>6☐ Traditional birth attendant<br>7☐ Health Development Army member	88○ Other (*Specify*)
**HSU24.**	During the delivery of your **last child**, did you experience any **serious health problems** related to BIRTH?	1○ Yes	0○ No SKIP TO HSU26
**HSU25.**	What **serious health problems** did you experience during the DELIVERY of your LAST child?<br><br>***INTERVIEWER*: Do NOT prompt. Select all that apply**	1☐ Bleeding<br>2☐ Severe headache<br>3☐ Blurred vision<br>4☐ Convulsions/fits<br>5☐ High fever<br>6☐ Loss of consciousness/fainting<br>7☐ Labour >12 hours	99○ Don't know<br>88☐ Other(*Specify*)

		8☐ Placenta not delivered 30min after delivery
**HSU26.**	How was your last child delivered?	1○ Caesarean section (belly cut open and baby taken out)<br>2○ Forceps/vacuum extraction<br>3○ Vaginal delivery<br>88○ Other (*Specify*)

4.3 Postpartum care

FOR WOMEN WHO HAD A LIVEBIRTH OR STILLBIRTH

Now I'd like to ask you some questions about after you gave birth to your last child

**HSU27**	After you gave birth to your **LAST child**, did someone check on your health?<br><br>*For example, someone examining you or asking you questions about your health?*	1○ Yes	0○ No SKIP TO HSU31
**HSU28**	How long after delivery did the **first** check take place?	☐☐ minutes<br>☐☐ hours<br>☐☐ days	☐☐ weeks<br>☐☐ months<br><br>0○ Do not remember
**HSU29**	Who checked on your health at that time?	1○ Doctor<br>2○ Nurse/midwife<br>3○ TBA<br>4○ Relative/friend	5○ HEW<br>88○ Other (*Specify*)
**HSU30**	Where did this **first** check-up take place?	1○ Own home<br>2○ Someone else's home (ex: relative, TBA)<br>3○ Government hospital<br>4○ Government health centre<br>5○ Government health post	6○ Private hospital<br>6○ Private clinic<br>88○ Other (*Specify*)
**HSU31**	During the **6 weeks after the birth** of your LAST baby, did you experience any **serious health problems** related to the birth?	1○ Yes	0○ No SKIP TO HSU35
**HSU32**	What problems did you experience?<br><br>***INTERVIEWER: Do NOT prompt. Select all that apply.***	1☐ Bleeding<br>2☐ Severe headache<br>3☐ Blurred vision<br>4☐ Convulsions/fits<br>5☐ Swollen face/hands<br>6☐ High fever<br>7☐ Loss of consciousness/fainting<br>8☐ Breathing difficulty<br>9☐ Severe weakness	10☐ Foul smelling vaginal discharge<br>11☐ Inability to control urine/stool<br>88☐ Other(Specify)

**HSU33**	Did you **get help/seek assistance** for the serious health problem(s) you experienced during the 6 weeks after the birth of your LAST child?	1○ Yes<br>0○ No SKIP TO HSU35 if REP14=livebirth<br>SKIP to HSU27B if REP14=abortion or miscarriage	
**HSU34**	Where did you get help from?<br>***INTERVIEWER: Select all that apply***	1☐ Husband<br>2☐ Family/relative<br>3☐ Friends/neighbours<br>4☐ Health extension worker<br>5☐ Doctor/nurse<br>6☐ Traditional birth attendant (TBA)	7☐ Health Development Army<br>88☐ Other (Specify)

FOR WOMEN WHO HAD A LIVEBIRTH ONLY

**HSU35**	SKIP TO MWA1 IF REP14=miscarriage, abortion or stillbirth<br>Did anyone check on **your baby's** health? For example, look at the cord to see if it was ok?	1○ Yes	0○ No SKIP TO HSU40
**HSU36**	How long after delivery did the **first** check on your baby take place?	☐☐ minutes<br>☐☐ hours<br>☐☐ days	☐☐ weeks<br>☐☐ months<br>0○ Do not remember
**HSU37**	Who checked on the baby's health at that time?	1○ Doctor<br>2○ Nurse/midwife<br>3○ TBA<br>4○ Relative/friend<br>5○ HEW	88○ Other (*Specify*)
**HSU38**	Where did this first check on the baby's health take place?	1○ Own home<br>2○ Someone else's home (ex: relative, TBA)<br>3○ Government hospital<br>4○ Government health centre<br>5○ Government health post	6○ Private hospital<br>6○ Private clinic<br>88○ Other (*Specify*)
**HSU40**	During the **first 28 days after birth**, did the baby experience any **serious health**	1○ Yes	0○ No SKIP TO MWA1

	**problems**?		
**HSU41**	What serious health problems did **the baby experience** during the first 28 days after birth?	1☐ Difficulty/fast breathing<br>2☐ Yellow skin/eyes<br>3☐ Poor feeding<br>4☐ Pus/blood around cord<br>5☐ Very small baby<br>6☐ Lesions or blisters<br>7☐ Convulsions (fits)<br>8☐ Loss of consciousness/weak<br>9☐ Red/swollen eyes with pus	10☐ Cold to touch<br>11☐ Vomiting/diarrhoea<br>12☐ Fever<br>88☐ Other(*Specify*)
**HSU42**	Did you get help/seek assistance for the baby's serious health problem(s)?	1○ Yes	0○ No SKIP TO MWA1
**HSU43**	Where did you get help from for the baby's health problems?<br>***Select all that apply***	1☐ Husband<br>2☐ Relative<br>3☐ Friends/neighbours<br>4☐ HEW<br>5☐ Doctor/Nurse<br>6☐ Traditional birth attendant	88☐ Other (*Specify*)

FOR WOMEN WHO HAD A MISCARRIAGE OR ABORTION

Now I'd like to ask you some questions about the care you received after your last pregnancy ended

**HSU27B**	After your **LAST pregnancy ended**, did someone check on your health?<br>*For example, someone examining you or asking you questions about your health?*	1○ Yes	0○ No SKIP TO HSU31B
**HSU28C**	How long after the LAST pregnancy ended did the **first** check take place?	☐☐ minutes<br>☐☐ hours<br>☐☐ days	☐☐ weeks<br>☐☐ months<br>0○ Do not remember
**HSU29**	Who checked on your health at that time?	1○ Doctor<br>2○ Nurse/midwife	5○ HEW<br>88○ Other (*Specify*)

		3○ TBA<br>4○ Relative/friend	
**HSU30**	Where did this **first** check-up take place?	1○ Own home<br>2○ Someone else's home (ex: relative, TBA)<br>3○ Government hospital<br>4○ Government health centre<br>5○ Government health post<br>6○ Private hospital<br>7○ Private clinic	88○ Other (*Specify*)
**HSU31B**	During the **6 weeks after** your LAST pregnancy ended, did you experience any **serious health problems** related to the pregnancy?	1○ Yes	0○ No SKIP TO MWA1
**HSU32B**	What problems did you experience?<br><br>*<u>INTERVIEWER</u>: Do NOT prompt. Select all that apply.*	1☐ Bleeding<br>2☐ Severe headache<br>3☐ Blurred vision<br>4☐ Convulsions/fits<br>5☐ Swollen face/hands<br>6☐ High fever<br>7☐ Loss of consciousness/fainting<br>8☐ Breathing difficulty<br>9☐ Severe weakness	10☐ Foul smelling vaginal discharge<br>11☐ Inability to control urine/stool<br>88☐ Other(Specify)
**HSU33B**	Did you **get help/seek assistance** for the serious health problem(s) you experienced during the **6 weeks** after your LAST pregnancy ended?	1○ Yes	0○ No SKIP TO MWA1
**HSU34B**	Where did you get help from?<br><br>***INTERVIEWER: Select all that apply***	1☐ Husband<br>2☐ Family/relative<br>3☐ Friends/neighbours<br>4☐ Health extension worker<br>5☐ Doctor/nurse<br>6☐ Traditional birth attendant (TBA)	7☐ Health Development Army<br>88☐ Other (Specify)

SECTION 5: [MWA] MATERNITY WAITING HOMES

I would like to now ask you some questions about maternity waiting homes

**MWA1.**	Have you **heard** of a maternity waiting home?	1○ Yes<br>0○ No<br>*INTERVIEWER:* Explain a maternity waiting home is a temporary place for pregnant women to stay close to the health centre before delivery for free if she lives far away or has health problems
**MWA2.**	Do you have a maternity waiting home **in** or **near** your kebele?	1○ Yes 0○ No 99○ Do not know
**MWA3.**	What sort of **services are offered/facilities available** at maternity waiting homes?<br><br>*PROBE: Any other?*<br><br>***Select all that apply***	**Facilities/amenities** 88☐ Other (*Specify*)<br>1☐ Beds for sleeping<br>2☐ Cooking facilities<br>3☐ Food and drinks<br>4☐ Toilet/latrine<br>5☐ Bathrooms<br>6☐ Electricity<br>7☐ Clean water<br>**Services**<br>8☐ Check up by nurse/midwife<br>9☐ Visits by HEWs<br>**Other**<br>9☐ Family visits allowed<br>10☐ Coffee ceremony
**MWA20**	What services and facilities do you think are **important to be available** to mothers who stay at maternity waiting homes?	**Facilities/amenities** 88☐ Other (*Specify*)<br>1☐ Beds for sleeping<br>2☐ Cooking facilities<br>3☐ Food and drinks<br>4☐ Toilet/latrine<br>5☐ Bathrooms<br>6☐ Electricity<br>7☐ Clean water<br>**Services**<br>8☐ Check up by nurse/midwife<br>9☐ Visits by HEWs<br>**Other**<br>9☐ Family visits allowed<br>10☐ Coffee ceremony

**MWA4.**	What **benefits** do you think maternity waiting homes offer?	0☐ None<br>1☐ Quick access to doctors/nurses<br>2☐ Chance for mothers to rest<br>3☐ No need to organize emergency transport for delivery<br>99○ Do not know<br>88☐ Other (*Specify*)
**MWA5.**	Do you know of anyone in your family/neighbourhood who has used a maternity waiting home?	1○ Yes 0○ No
**MWA6.**	Have you **ever visited** someone at a maternity waiting home?	1○ Yes 0○ No
**MWA7.**	Have you **ever used** a maternity waiting home yourself?	1○ Yes SKIP TO MWH18 0○ No
**MWA17.**	What are the reasons for **never having used** a maternity waiting home?	1☐ Did not know about maternity waiting homes<br>2☐ Do not see any benefit of staying at maternity waiting homes<br>3☐ Was not sure when to go to the maternity waiting home / was not sure of delivery date<br>4☐ Did not plan for delivery<br>5☐ Did not want to give birth at the health centre<br>6☐ Live close to health facility where I can deliver<br>7☐ Too costly to get to maternity waiting homes<br>8☐ Poor services – no food, etc<br>9☐ Have no childcare<br>10☐ Husband/family did not want me to stay there<br>11☐ Did not get a referral<br>12☐ Have heard of negative experiences from women who have stayed there before<br>13☐ There was no one to take me to the maternity waiting home<br>14☐ I did not have family/friends available to look after me at the maternity waiting home<br>15☐ Inadequate sleeping arrangements ex: no

		blankets<br>[16]☐ Health centre staff do not look after women/ check on them<br>[17]☐ No toilets<br>[18]☐ No bathrooms/bathing areas<br>[19]☐ Inadequate lighting / no power sources<br>[20]☐ Family is not allowed to stay with me<br>[21]☐ No food served or adequate kitchen facilities<br>[22]☐ No clean water available<br>[23]☐ No entertainment/activities available<br>[24]☐ Did not have a positive experience during previous stay<br>[88]☐ Other (*Specify*)
**MWA18.**	Did you use a maternity waiting home during your **last pregnancy**?	[1]○ Yes SKIP TO MWA8 [0]○ No
**MWA19.**	What were the reasons for **not using** a maternity waiting home during your **last pregnancy**?<br><br>*INTERVIEWER: Do NOT read out. Select all that apply.*	[1]☐ Did not know about maternity waiting homes<br>[2]☐ Do not see any benefit of staying at maternity waiting homes<br>[3]☐ Was not sure when to go to the maternity waiting home / was not sure of delivery date<br>[4]☐ Did not plan for delivery<br>[5]☐ Did not want to give birth at the health centre<br>[6]☐ Live close to health facility where I can deliver<br>[7]☐ Too costly to get to maternity waiting homes<br>[8]☐ Poor services – no food, etc<br>[9]☐ Have no childcare<br>[10]☐ Husband/family did not want me to stay there<br>[11]☐ Did not get a referral<br>[12]☐ Have heard of negative experiences from women who have stayed there before<br>[13]☐ There was no one to take me to the maternity waiting home<br>[14]☐ I did not have family/friends available to look after me at the maternity waiting home<br>[15]☐ Inadequate sleeping arrangements ex: no blankets<br>[16]☐ Health centre staff do not look after women/ check on them

		17☐ No toilets<br>18☐ No bathrooms/bathing areas<br>19☐ Inadequate lighting / no power sources<br>20☐ Family is not allowed to stay with me<br>21☐ No food served or adequate kitchen facilities<br>22☐ No clean water available<br>23☐ No entertainment/activities available<br>24☐ Did not have a positive experience during previous stay<br>88☐ Other (*Specify*)
**MWA8.**	Were you **referred** to the maternity waiting home by a HEW or other health worker?	1○ Yes 0○ No
**MWA9.**	SKIP TO MWA15 IF MWA18=No<br><br>Why did you decide to go to the maternity waiting home?<br><br>*<u>INTERVIWER</u>: Do NOT read out. Select all that apply.*	1☐Referred by HEW<br>2☐ Used MWH during previous pregnancy<br>3☐ Live far away from facility<br>4☐ Expecting complications during delivery<br>5☐ Not satisfied with TBA/ want to deliver at health facility<br>6☐ Needed rest<br>7☐ Husband/family wanted me to<br>88☐ Other (*Specify*)
**MWA10.**	SKIP TO MWA15 IF MWA18=No<br><br>How **close to your delivery date** were you when you went to the maternity waiting home?<br><br>*INTERVIEWER: Explain to woman that you need to know how much time was left before she gave birth.*	☐☐ days ☐☐ weeks

**MWA11.**	SKIP TO MWA15 IF MWA18=No<br><br>How long did you stay at the maternity waiting home **before** delivery?	☐☐ days ☐☐ weeks
**MWA12.**	SKIP TO MWA15 IF MWA18=No<br><br>How long did you stay at the maternity waiting home **after** delivery?	☐☐ days ☐☐ weeks
**MWA13.**	SKIP TO MWA15 IF MWA18=No<br><br>What services were available to you during your stay at the maternity waiting home?<br><br>*INTERVIEWER: Do NOT read out. Select all that apply.*	**Facilities/amenities**<br>1☐ Beds for sleeping<br>2☐ Cooking facilities<br>3☐ Food and drinks<br>4☐ Toilet/latrine<br>5☐ Bathrooms<br>6☐ Electricity<br>7☐ Clean water<br>**Services**<br>8☐ Check up by nurse/midwife<br>9☐ Visits by HEWs<br>**Other**<br>10☐ Family visits allowed<br>11☐ Coffee ceremony<br>88☐ Other (*Specify*)
**MWA15**	Would you recommend using a maternity waiting home to your pregnant family and/or friends?	1○ Yes SKIP TO WDK1 0○ No
**MWA16**	Why would you **NOT recommend** the use of a maternity waiting home to your pregnant family members or friends?	1☐ Culturally appropriate to deliver at home<br>2☐ MWHs are expensive<br>3☐ No privacy/confidentiality at MWHs<br>4☐ TBAs are closer than MWHs<br>4☐ Poor services/inadequate supplies<br>88☐ Other (*Specify*)

SECTION 6: [WDK] DANGER SIGN KNOWLEDGE & PERCEPTIONS

I would now like to ask you some questions about pregnancy and childbirth in general.

**WDK1.**	Can women experience **serious, unexpected health problems** during pregnancy?	1 ○ Yes	0 ○ No SKIP TO WDK5	99 ○ Do not know SKIP TO WDK5
**WDK2.**	What are some of the serious health problems that can occur during **pregnancy**?<br><br>*INTERVIEWER: **Do NOT prompt. Select all mentioned.***	1 ☐ Bleeding<br>2 ☐ Severe headache<br>3 ☐ Blurred vision<br>4 ☐ Convulsions/fits<br>5 ☐ Swollen face/hands<br>6 ☐ High fever<br>7 ☐ Loss of consciousness/fainting<br>8 ☐ Breathing difficulty<br>9 ☐ Severe weakness<br>10 ☐ Severe abdominal pain<br>11 ☐ More/less fetal movement<br>12 ☐ Water breaks without labour<br>13 ☐ Persistent vomiting<br>14 ☐ Infection	15 ☐ Mental health problems (ex: depression)<br>88 ☐ Other(*Specify*)	
**WDK3.**	In your opinion could a pregnant woman **die** from this problem/any of these problems?	1 ○ Yes	0 ○ No	99 ○ Do not know
**WDK4.**	In your opinion could a pregnant woman **lose the baby** from this problem/any of these problems?	1 ○ Yes	0 ○ No	99 ○ Do not know
**WDK5.**	Can a woman experience serious health problems during **labour/childbirth**?	1 ○ Yes	0 ○ No SKIP TO WDK9	99 ○ Do not know SKIP TO WDK9
**WDK6.**	What are some of the serious health problems that can occur during **labour/childbirth**?<br><br>*INTERVIEWER: **Do NOT prompt. Select all mentioned.***	1 ☐ Bleeding<br>2 ☐ Severe headache<br>3 ☐ Blurred vision<br>4 ☐ Convulsions/fits<br>5 ☐ High fever<br>6 ☐ Loss of consciousness/fainting<br>7 ☐ Labour > 12 hours	8 ☐ Placenta not delivered 30min after birth<br>99 ☐ Do not know<br>88 ☐ Other (*Specify*)	

**WDK7.**	In your opinion could a woman **die** from this problem/any of these problems?	1○ Yes	0○ No	99○ Do not know
**WDK8.**	In your opinion could a woman **lose her baby** from this problem/any of these problems?	1○ Yes	0○ No	99○ Do not know
**WDK9.**	Can a woman experience serious health problems during the **first 6 weeks after delivery**?	1○ Yes	0○ No SKIP TO WDK12	99○ Do not know SKIP TO WDK12
**WDK10.**	What are some of the serious health problems that can occur in the mother during the **first 6 weeks after delivery**?<br><br>*INTERVIEWER:* ***Do NOT prompt. Select all mentioned.***	1☐ Severe bleeding<br>2☐ Severe headache<br>3☐ Blurred vision<br>4☐ Convulsions<br>5☐ Swollen face/hands<br>6☐ High fever<br>7☐ Loss of consciousness<br>8☐ Breathing difficulty<br>9☐ Severe weakness<br>10☐ Foul smelling vaginal discharge<br>11☐ Inability to control urine/stool	88☐ Other(*Specify*)	
**WDK11.**	In your opinion could a woman **die** from this problem/any of these problems?	1○ Yes	0○ No	99○ Do not know
**WDK12.**	Can a baby experience serious health problems during the **first 28 days of life/after birth?**	1○ Yes	0○ No SKIP TO ATT1	99○ Do not know SKIP TO ATT1
**WDK13.**	What are some of the serious health problems that can occur in the baby during the **first 28 days of life/after birth**?<br><br>*INTERVIEWER:* ***Do NOT prompt. Select all mentioned.***	1☐ Difficulty/fast breathing<br>2☐ Yellow skin/eyes<br>3☐ Poor feeding<br>4☐ Pus/blood around cord<br>5☐ Very small baby<br>6☐ Lesions or blisters<br>7☐ Convulsions (fits)<br>8☐ Loss of consciousness/weak	9☐ Red/swollen eyes with pus<br>10☐ Cold to touch<br>11☐ Vomiting/diarrhoea<br>12☐ Fever<br>88☐ Other(*Specify*)	

**WDK14.**	In your opinion could a **baby die** from this problem/any of these problems?	1○ Yes	0○ No	99○ Do not know

SECTION 7: [ATT] ATTITUDES TOWARDS MATERNAL CARE SERVICES

Now I am going to read out a list of common opinions about pregnancy, delivery and the period after childbirth. Please tell me if you agree, disagree or neither agree nor disagree with these statements. There is no right or wrong answer. We are only interested in hearing your opinion about the situation in your kebele.

**ATT1.**	A woman should **plan ahead** of time where she will give birth to her baby	1○ Agree	2○ Neither agree nor disagree	3○ Disagree
**ATT2.**	**Antenatal care visits** at the health facility are necessary for healthy pregnant women	1○ Agree	2○ Neither agree nor disagree	3○ Disagree
**ATT3.**	It is not necessary for a husband to accompany his wife to antenatal care visits	1○ Agree	2○ Neither agree nor disagree	3○ Disagree
**ATT4.**	Women who are **healthy are not at risk** for complications during delivery	1○ Agree	2○ Neither agree nor disagree	3○ Disagree
**ATT5.**	When women do not go to a health facility to give birth it is often because it is **too expensive**	1○ Agree	2○ Neither agree nor disagree	3○ Disagree
**ATT6.**	When women do not go to a health facility to give birth it is often because it is **too difficult to get there**	1○ Agree	2○ Neither agree nor disagree	3○ Disagree
**ATT7.**	When women do not go to a health facility to give birth it is often because the **staff do not treat women respectfully**	1○ Agree	2○ Neither agree nor disagree	3○ Disagree
**ATT8.**	It is not necessary for **a husband to accompany his wife** to the health facility when she is giving birth.	1○ Agree	2○ Neither agree nor disagree	3○ Disagree
**ATT9.**	When women do not go to a health facility to give birth it is often because they are afraid of **receiving unwanted procedures**	1○ Agree	2○ Neither agree nor disagree	3○ Disagree
**ATT10.**	When women do not go to a health facility to give birth it is often because it is **unacceptable to be attended by male health workers**	1○ Agree	2○ Neither agree nor disagree	3○ Disagree
**ATT11.**	Once a woman has had experience delivering a baby, she does not need to go to a health facility to deliver any of her future babies	1○ Agree	2○ Neither agree nor disagree	3○ Disagree

**ATT12.**	A woman who has **complications** during delivery/labour has a better chance of survival if doctors/nurses/midwives are present	1○ Agree	2○ Neither agree nor disagree	3○ Disagree
**ATT13.**	A **newborn** is most vulnerable to death and illness between **birth and 28 days** after birth	1○ Agree	2○ Neither agree nor disagree	3○ Disagree
**ATT14.**	It is important for mothers who have recently given birth to visit a health facility with their newborn for a check up	1○ Agree	2○ Neither agree nor disagree	3○ Disagree
**ATT15.**	It is important to get **babies vaccinated** at the health facility to protect them against common illnesses and possible death	1○ Agree	2○ Neither agree nor disagree	3○ Disagree

SECTION 8: [PHF] PERCEPTIONS OF HEALTH FACILITIES

I would now like to ask you some questions about places where women give birth and receive care during pregnancy

**PHF1.**	Can you tell me where a woman can go to give birth to a baby with the assistance from a doctor/nurse/midwife?<br><br>*INTERVIEWER*: ***Do NOT prompt. Select all mentioned.***	1☐ Hospital<br>2☐ Health centre<br>3☐ Health post<br>4☐ Maternity waiting home<br>88☐ Other (*Specify*)
**PHF2.**	Do women have to **pay to receive services** related to pregnancy and delivery at this place?	1○ Yes<br>0○ No SKIP PHF4<br>99○ Do not know SKIP PHF4
**PHF3.**	Would you say the amount to be paid is expensive, reasonable, or cheap?	1○ Expensive<br>2○ Reasonable<br>3○ Cheap
**PHF4.**	Is there a health facility **in or near** your kebele?	1○ Yes<br>0○ No SKIP TO PHF10<br>99○ Do not know SKIP TO PHF10
**PHF5.**	What type of a health facility is it?<br>***INTERVIEWER: Read out answer options if necessary***	1○ Health post<br>2○ Health centre<br>3○ Government Hospital<br>5○Private hospital<br>4○ Private clinic<br>88○ Other (*Specify*)
**PHF6.**	How would you usually get there?<br><br>***PROBE: What type of transport would you mainly use?***	1○ By foot<br>2○ By taxi<br>3○ Bajaj (motorbike rickshaw)<br>4○ Local stretcher<br>5○ Ambulance<br>6○ Horse/mule<br>7○ Bicycle<br>88○ Other (*Specify*)

**PHF7.**	**How long** does it take to get to this facility from your home using this method of transport?	☐☐ minutes	☐☐ hours ⁰○ Do not remember
**PHF8**	In your opinion, **how would you rate the services** offered at the health facility in/near your kebele?<br><br>Would you say they are very good, good, fair, poor or very poor?	¹○ Very good<br>²○ Good<br>³○ Fair<br>⁴○ Poor<br>⁵○ Very poor	
**PHF9**	Can you tell me what is/are the reason(s) that you feel that way about this health facility?	1☐ Doctor/nurse always there<br>2☐ Facility always open<br>3☐ Respectful staff<br>4☐ Short waiting times<br>5☐ Competent staff<br>5☐ Patient privacy maintained<br>88☐ Other (*Specify*)	6☐ Doctor/nurse never there<br>7☐ Facility often closed<br>8☐ Disrespectful staff<br>9☐ Long waiting times<br>10☐ Incompetent staff<br>11☐ Patient privacy not maintained<br>89☐ Other (*Specify*)

ONLY FOR WOMEN WHO GAVE BIRTH TO THEIR LAST CHILD AT A HEALTH FACILITY (SEE HSU17)

**PHF10**	What health facility did you deliver your LAST child at?	**GOMMA**<br>1○ Beshasha<br>2○ Chami Chago<br>3○ Choche<br>4○ Dhayi Kechene<br>5○ Gembe<br>6○ Kedemasa<br>7○ Limu Shayi<br>8○ Meti Koticha<br>9○ Omo Gurude<br>10○ Yachi<br>88○ Other (*Specify*)	**SEKA CHEKORSA**<br>1○ Bake Gudo<br>2○ Buyo Kechema<br>3○ Dabo Yaya<br>4○ Detu Kersu<br>5○ Geta Bake<br>6○ Lilu Omoti<br>7○ Seka<br>8○ Setemma<br>9○ Wokito<br>88○ Other (*Specify*)	**KERSA**<br>1○ Adere Dika<br>2○ Bala Wajo<br>3○ Bulbul<br>4○ Kusaye Beru<br>5○ Kara Gora<br>6○ Kellacha<br>7○ Serbo<br>88○ Other (*Specify*)

**PHF11**	How did you get to this facility?	1○ By foot<br>2○ By taxi<br>3○ Bajaj (motorbike rickshaw)<br>4○ Local stretcher<br>5○ Ambulance<br>6○ Horse/mule<br>7○ Bicycle	88○ Other (*Specify*)
**PHF12**	**How long** does it take to get to this facility from your home using this method of transport?	☐☐ minutes	☐☐ hours
**PHF13**	In your opinion, **how would you rate the services** offered at this health facility?<br><br>Would you say they are very good, good, fair, poor or very poor?	1○ Very good<br>2○ Good<br>3○ Fair<br>4○ Poor<br>5○ Very poor	
**PHF14**	Can you tell me what is/are the reason(s) that you feel that way about this health facility?<br>***Do NOT read out. Select all that apply.***	1☐ Doctor/nurse always there<br>2☐ Facility always open<br>3☐ Respectful staff<br>4☐ Short waiting times<br>5☐ Competent staff<br>5☐ Patient privacy maintained<br>88☐ Other (*Specify*)	6☐ Doctor/nurse never there<br>7☐ Facility often closed<br>8☐ Disrespectful staff<br>9☐ Long waiting times<br>10☐ Incompetent staff<br>11☐ Patient privacy not maintained<br>89☐ Other (*Specify*)

SECTION 9: [QOL] HEALTH-RELATED QUALITY OF LIFE ASSESSMENT

I would like to now ask you a few questions about your health-related quality of life. I will read out a statement that describes some a situation that might be affecting your quality of life, please tell me the extent to which this is true.

**QOL1.**	Problems in walking about	1○ No problem<br>2○ Some problems<br>3○ Confined to bed
**QOL2.**	Problems washing or dressing yourself	1○ No problem<br>2○ Some problems<br>3○ Unable to do

**QOL3.**	Problems doing usual activities like housework, farming, etc	1○ No problem<br>2○ Some problems<br>3○ Unable to do
**QOL4.**	Feel pain or discomfort	1○ No pain/discomfort<br>2○ Moderate pain/discomfort<br>3○ Extreme pain/discomfort
**QOL5.**	Feel anxious or depressed	1○ No anxiety/depression<br>2○ Moderate anxiety/depression<br>3○ Extreme anxiety/depression
**QOL6.**	On a scale from 0 to 10, where 10 is the best health you can imagine and 0 is the worst health you can imagine, could you please tell me how good or bad your health is TODAY.	☐☐

SECTION 10: [SS] SOCIAL SUPPORT

I would like to ask some questions about social support that you received during pregnancy, during delivery and after delivery.

**SS1.**	During your LAST pregnancy was there someone that you could depend on when you were in need?	1○ Yes	0○ No SKIP TO SS3
**SS2.**	What was the relationship of this person/these people to you?<br><br>***Do NOT read out. Select all that apply.***	1☐ Husband<br>2☐ Parents<br>3☐ Siblings<br>4☐ Friends/Neighbours<br>5☐ In-laws<br>6☐ Religious group<br>7☐ Community group<br>8☐ Health worker<br>9☐ Health extension worker	88☐ Other (*Specify*)
**SS3.**	For your LAST child, did you receive any **practical help** from anyone?<br><br>*Example: help with child care, house chores, food preparation, cattle herding, etc*	1○ Yes	0○ No SKIP TO SS6
**SS4.**	When did you receive this help?<br>***Select all that apply***	1☐ During pregnancy 2☐ during delivery period	3☐ after delivery
**SS5.**	From whom did you receive this help?	1☐ Husband<br>2☐ Parents<br>3☐ Siblings<br>4☐ Friends/Neighbours<br>5☐ In-laws<br>6☐ Religious group	88☐ Other (*Specify*)

		7☐ Community group<br>8☐ Health worker<br>9☐ Health extension worker
**SS16**	How close is your relationship with the people that you received the assistance from?	0○ Not strong<br>1○ Strong<br>2○ Very strong<br>99○ Do not know
**SS17**	How satisfied were you with the support/help you received?	0○ Not satisfied<br>1○ Satisfied<br>2○ Very satisfied<br>99○ Do not know
**SS6.**	For your LAST child, was there anyone who **accompanied you** to the health facility and provided personal assistance to you?<br><br>*Example: helped you bathe, stayed with you at the health facility, escorted you to the latrine at night, etc*	1○ Yes<br>0○ No SKIP TO SS9
**SS7.**	When did you receive this help?<br>***Select all that apply***	1☐ During pregnancy<br>2☐ during delivery period<br>3☐ after delivery
**SS8.**	What was the **relationship** of the person/people you received this assistance from?	1☐ Husband<br>2☐ Parents<br>3☐ Siblings<br>4☐ Friends/Neighbours<br>5☐ In-laws<br>6☐ Religious group<br>7☐ Community group<br>8☐ Health worker<br>9☐ Health extension worker<br>88☐ Other (*Specify*)
**SS18**	How close is your relationship with the people that you received the assistance from?	0○ Not strong<br>1○ Strong<br>2○ Very strong<br>99○ Do not know
**SS19**	How satisfied were you with the support/help you received?	0○ Not satisfied<br>1○ Satisfied<br>2○ Very satisfied<br>99○ Do not know
**SS9.**	For your LAST child, was there anyone who provided you with **emotional support**?<br><br>*Example: someone who you could talk to when*	1○ Yes<br>0○ No SKIP TO SS12

	you were upset or worried, provided you with encouragement and reassurance	
**SS10.**	When did you receive this help?<br>***Select all that apply***	1☐ During pregnancy 2☐ during delivery period 3☐ after delivery
**SS11.**	What was the relationship of the person/people you received this assistance from?	1☐ Husband<br>2☐ Parents<br>3☐ Siblings<br>4☐ Friends/Neighbours<br>5☐ In-laws<br>6☐ Religious group<br>7☐ Community group<br>8☐ Health worker<br>9☐ Health extension worker<br>88☐ Other (*Specify*)
**SS20**	How close is your relationship with the people that you received the assistance from?	0○ Not strong<br>1○ Strong<br>2○ Very strong<br>99○ Do not know
**SS21**	How satisfied were you with the support/help you received?	0○ Not satisfied<br>1○ Satisfied<br>2○ Very satisfied<br>99○ Do not know
**SS12.**	For your last child, did you receive any **financial or in-kind assistance** from anyone?<br>*For example: received money/loans, medicines, clothes, food, etc*	1○ Yes 0○ No SKIP TO SS15
**SS13.**	What did you receive?<br>***Select all that apply***	1☐ Money<br>2☐ Food<br>3☐ Clothes<br>4☐ Medicine<br>5☐ Cooking fuel<br>6☐ Furniture<br>88☐ Other (*Specify*)
**SS14.**	When did you receive this help?<br>***Select all that apply***	1☐ During pregnancy 2☐ during delivery period 3☐ after delivery
**SS15.**	What was the relationship of the person/people you received this assistance from?	1☐ Husband<br>2☐ Parents<br>3☐ Siblings<br>4☐ Friends/Neighbours<br>5☐ In-laws<br>6☐ Religious group<br>7☐ Community group<br>8☐ Health worker<br>9☐ Health extension<br>88☐ Other (*Specify*)

		worker
**SS22**	How close is your relationship with the people that you received the assistance from?	⁰O Not strong ²O Very strong<br>¹O Strong ⁹⁹O Do not know
**SS23**	How satisfied were you with the support/help you received?	⁰O Not satisfied ²O Very satisfied<br>¹O Satisfied ⁹⁹O Do not know

SECTION 11: [DEM] SOCIODEMOGRAPHIC INFORMATION continued

Thank you. We are now going to end with some final questions on your household.

**DEM21.**	Do you **own this or any other house** either alone or jointly with someone else?	0 O No 1 O Alone only 2 O Jointly
**DEM22.**	Does any member of this household own any **agricultural land**?<br>*Ex: land where you grow coffee, teff, maize, etc*	1 O Yes 0 O No 99 O Do not know
**DEM24**	Does any member of this household own any **non-agricultural land**?<br>*Ex: land where cattle graze, etc*	1 O Yes 0 O No 99 O Do not know
**DEM26**	What is the main source of **drinking water** for members of your household?	1 O Piped into dwelling<br>2 O Piped into yard/plot<br>3 O Public tap/standpipe<br>4 O Borehole<br>5 O Protected well<br>6 O Unprotected well<br>7 O Protected spring<br>8 O Unprotected spring<br>9 O Rainwater<br>88 O Other (*Specify*)
**DEM29**	What kind of **toilet facility** do members of your household usually use?	0 O No facility/use bush<br>1 O Flush to piped sewer<br>2 O Flush to septic tank<br>3 O Flush to pit latrine<br>4 O Flush, don't know<br>5 O Ventilated improved pit latrine<br>6 O Pit latrine with slab<br>7 O Open pit<br>88 O Other (*Specify*)
**DEM30**	What type of **fuel** does your household mainly use for **cooking**?	1 O Electricity<br>2 O Natural gas<br>3 O Kerosene<br>4 O Charcoal<br>5 O Wood<br>88 O Other (*Specify*)
**DEM32**	How many **rooms** in this household are used for **sleeping**?	☐☐
**DEM34**	Does this household own any livestock/farm animals or poultry?	1 O Yes 0 O No SKIP TO DEM36

**DEM35**	How many of the following **animals** does the household own?	[a]☐ Milk cows<br>[b]☐ Bulls/ox<br>[c]☐ Horses/donkeys/mules<br>[d]☐ Goats<br>[e]☐ Sheep<br>[f]☐ Chicken/poultry<br>[g]☐ Other (*Specify*)<br>[g]☐ Other (*Specify*)	☐☐<br>☐☐<br>☐☐<br>☐☐<br>☐☐<br>☐☐<br>☐☐<br>☐☐
**DEM36**	Does your household have any of the following?	[a]☐ Electricity…………………………<br>[b]☐ Radio……………………………….<br>[c]☐ Television……………….……….<br>[d]☐ Refrigerator……………………<br>[e]☐ Car/truck……………………….<br>[f]☐ Bicycle……………..……………..<br>[g]☐ Motorcycle…………………….	[1]☐ Yes [0]☐ No<br>[1]☐ Yes [0]☐ No<br>[1]☐ Yes [0]☐ No<br>[1]☐ Yes [0]☐ No<br>[1]☐ Yes [0]☐ No<br>[1]☐ Yes [0]☐ No<br>[1]☐ Yes [0]☐ No
**DEM38A**	Do you know how much the total income for your household was last year?<br><br>*<u>INTERVIEWER</u>: Assist women to provide a rough estimate of household income i.e income earned by all adult household members from formal and informal sources*	[1]○ Yes	[0]○ No
**DEM38**	Last year, what was the total income for **your household** for the year?	☐☐☐☐☐☐ Birr [99]○ Do not know	
**DEM46**	Do you have health insurance?	[1]○ Yes [0]○ No	
**DEM47**	What type of health insurance is it?<br>***Prompt if necessary***	[1]○ Government<br>[2]○ Private	[88]○ Other(*Specify*)
**DEM48**	INTERVIEWER: Observe and record<br><br>Main material of the **floor** of the dwelling	[1]○ Earth/sand<br>[2]○ Dung<br>[3]○ Wood planks<br>[4]○ Polished wood<br>[5]○ Cement	[6]○ Bricks<br>[88]○ Other (*Specify*)
**DEM49**	INTERVIEWER: Observe and record<br><br>Main material of the **roof** of the dwelling	[1]○ Thatch<br>[2]○ Corrugated iron<br>[3]○ Wood<br>[4]○ Cement	[88]○ Other (*Specify*)

**DEM50**	INTERVIEWER: Observe and record<br><br>Main material of the **exterior walls** of the dwelling	1○ Mud with wood<br>2○ Stone with mud<br>3○ Wood<br>4○ Cement<br>5○ Bricks<br>88○ Other (*Specify*)

Thank you for taking the time to share this information with us.
